# Optical-OFDM VLC System: Peak-to-Average Power Ratio Enhancement and Performance Evaluation

**DOI:** 10.3390/s24102965

**Published:** 2024-05-07

**Authors:** Yasser A. Zenhom, Ehab K. I. Hamad, Mohammed Alghassab, Mohamed M. Elnabawy

**Affiliations:** 1 Communication Department, Modern Academy for Engineering and Technology, Cairo 11571, Egypt; m.elnabawy@ieee.org; 2 Electrical Engineering Department, Faculty of Engineering, Aswan University, Aswan 81542, Egypt; e.hamad@aswu.edu.eg; 3 Electrical Engineering Department, College of Engineering, Shaqra University, Riyadh 11911, Saudi Arabia

**Keywords:** VLC, MCM, LACO-OFDM, ASCO-OFDM, PAPR

## Abstract

Visible Light Communication (VLC) systems are favoured for numerous applications due to their extensive bandwidth and resilience to electromagnetic interference. This study delineates various constructions of Optical Orthogonal Frequency Division Multiplexing (O-OFDM) approaches employed in VLC systems. Various factors are elaborated within this context to ascertain a more effective O-OFDM approach, including constellation size, data arrangement and spectral efficiency, power efficiency, computational complexity, bit error rate (BER), and peak-to-average power ratio (PAPR). This paper seeks to assess these approaches’ BER and PAPR performance across varying modulation orders. Regrettably, in VLC systems based on OFDM methodology, the superposition of multiple subcarriers results in a high PAPR. Therefore, this study aims to diminish the PAPR in VLC systems, enhancing system performance. We propose a non-distorting PAPR reduction technique, namely the Vandermonde-Like Matrix (VLM) precoding technique. The suggested technique is implemented across various O-OFDM approaches, including DCO-OFDM, ADO-OFDM, ACO-OFDM, FLIP-OFDM, ASCO-OFDM, and LACO-OFDM. Notably, this method does not affect the system’s data rate because it does not require the mandatory transmission of side information. Furthermore, this technique can decrease the PAPR without impacting the system’s BER performance. This study compares the proposed PAPR reduction technique against established methods documented in the literature to evaluate their efficacy and validity rigorously.

## 1. Introduction

The proliferation of smartphones and the Internet of Things (IoT) has led to a paradigm shift in communication requirements, necessitating high-throughput and low-latency networks to facilitate real-time applications and seamless connectivity. Wireless Fidelity (WiFi) is becoming a common method of offering rapid internet and network connections in households and businesses. The well-known issue of spectrum crunch, arising from the shortage of usable natural frequency spectrum in wireless networks, necessitates solutions for developing next-generation communication systems. Based on the ITU’s global forecast for mobile data traffic, mobile data usage is anticipated to increase by approximately 55% annually from 2020 to 2030. This growth is expected to reach 607 exabytes (EB) by 2025 and an astounding 5016 EB by 2030 [1]. As a result, it is critical to make optimum use of the spectrum in order to support the growing number of high-data-rate devices. Visible Light Communication (VLC) has emerged as a viable and promising solution to mitigate the limitations inherent in conventional wireless network technologies [2,3].

VLC technology is widely used for precise positioning, the fast streaming of videos, data broadcasting indoors, and underwater transmission. It is also common in vehicle communication and electromagnetic interference-sensitive environments like aviation and hospitals, supporting music and audio signals with 3GPP audio coders [4,5,6,7]. VLC, in conjunction with intensive wavelength division multiplexing, is used in cutting-edge communication technologies such as 6G mobile communication systems [1].

VLC employs an intensity modulation and direct detection (IM/DD) scheme to transmit data at speeds that surpass the persistence of the human eye by modulating the intensity of Light-Emitting Diodes (LEDs) [8]. Compared to traditional Radio Frequency (RF) technology, VLC offers several advantages, such as data transfer with higher speeds, security enhancement, access to a broader, unregulated bandwidth, remarkable efficiency in terms of energy consumption, and comparatively cost-effective infrastructure [9].

One of the most prevalent applications of LED technology in VLC systems is its unique ability to serve both as a source of illumination and a means of high-speed data transmission [2]. This dual functionality presents two primary challenges: addressing the flickering effect and achieving effective dimming control. Furthermore, modern lighting devices now offer dimming capabilities, allowing for precise control of brightness levels. Given the potential for energy preservation, brightness control is a vital characteristic in the context of VLC. As a result, selecting proper modulation formats becomes critical in order to efficiently solve the difficulties of flicker reduction, dimming control, and providing reliable high-speed data connection [10]. 

In the realm of VLC, modulation techniques can be broadly organized into single-carrier modulation (SCM) and multicarrier modulation (MCM) techniques [4]. SCM techniques like analog pulse modulation types and on-off keying are employed to construct VLC systems, resulting in lower data rates. To mitigate this drawback, MCM techniques, such as O-OFDM, have been introduced [9,11]. O-OFDM modulation offers a promising approach to enhancing the data transmission capacity of LEDs by effectively utilizing a wider range of light frequencies, enabling faster data communication. Consequently, various modified O-OFDM systems have been explored, considering factors such as spectral efficiency, power efficiency, computational complexity, BER, and LED nonlinearity [12]. The optical-OFDM depends on the DC bias, also known as the DCO-OFDM approach, which has the benefit of obtaining the maximum data rate in VLC systems. It achieves higher data rates at the cost of applying a DC bias to transform the output bipolar samples into unipolar samples. Nonetheless, this leads to ineffective power utilization and influences the system’s BER performance [13].

Conversely, the FLIP-OFDM and unipolar OFDM techniques employ the transmit real output in two sequential frames without relying on DC bias for unipolar conversion. Consequently, these schemes feature a lower data rate but exhibit superior BER performance compared to the DCO-OFDM system [14]. Asymmetrically clipped optical ACO-OFDM utilizes the clipping of negative samples for unipolar conversion, eliminating the need for a DC bias. This approach enables the transmission of real unipolar output samples in a single frame, similar to DCO-OFDM, but with improved BER performance. ACO-OFDM shares the same data rate as FLIP-OFDM and U-OFDM [15]. Asymmetrically clipped DC-biased optical ADO-OFDM can be viewed as a hybrid technique, combining elements from both DCO and ACO-OFDM. While ADO-OFDM maintains the same data rate as DCO-OFDM, it exhibits inferior BER performance compared to previously mentioned approaches [16,17]. 

The asymmetrically and symmetrically clipping optical ASCO-OFDM technique is a hybrid approach. This approach can achieve a higher data rate with acceptable BER performance. This approach offers a higher data rate than ACO-OFDM and FLIP-OFDM while maintaining an acceptable BER performance compared to DCO-OFDM [18]. The layered asymmetrically clipped optical LACO-OFDM scheme also presents different data rates depending on its structural configuration, leading to varying BER performance. Specifically, LACO-OFDM with a two-layer structure matches the data rate of ASCO-OFDM, and as the number of layers increases, the output data rate also increases. A comprehensive explanation of these O-OFDM schemes will be provided in this paper [19].

Combining parallel data streams to create the OFDM or O-OFDM signal introduces the challenge of PAPR [3]. Top-of-form high PAPR is an inherent challenge of OFDM, leading to nonlinear and distorted signals as well as increased strength demands for the transmitter amplifier [20]. This issue becomes particularly critical in the context of VLC, where LEDs act as transmitter elements. LEDs can be damaged by high-power signals because they operate within a limited voltage range and possess a non-linear voltage-to-current (V-I) relationship. The nonlinear (V-I) properties of LEDs contribute to distortion in the O-OFDM signal, which is exacerbated by its high PAPR. The intense peaks in the OFDM signal can cause LED chips to overheat. Consequently, prior to sending the O-OFDM signal via the LEDs, it is crucial to mitigate its high PAPR [21]. In this study, a PAPR reduction approach is used to solve the limited dynamic range of LEDs. To optimize the performance of the O-OFDM systems. This paper introduces the utilization of the Vandermonde-Like matrix (VLM) pre-coding technique for various MCM methods previously mentioned. This precoding method minimizes PAPR effectively without sacrificing BER performance and operates without the need for supplementary side details or additional processing [21].

The novelty of this work can be summarized in four key aspects. Firstly, as far as the authors are aware, this is the first comprehensive exposition of various MCM techniques employed in VLC communication systems. This exposition encompasses detailed insights into transmitter and receiver structures, spectral efficiency, symbol arrangement, power efficiency, and computational complexity. Second, in the field of lowering the PAPR for VLC systems, this study is the first to introduce a PAPR reduction approach especially designed for O-OFDM systems. Third, the study conducts a thorough evaluation of PAPR and BER performance for these MCM techniques across different modulation orders. Fourth, it includes a comprehensive description of the PAPR reduction technique, along with the quantification of its efficacy in reducing PAPR for these MCM techniques. Additionally, the research assesses the impact of this reduction technique on BER performance.

The arrangement of this paper is as follows: Section 2 delineates the O-OFDM system models and parameters. Section 3 outlines the criteria used to assess the effectiveness of MCM approaches. Section 4 details PAPR reduction methodologies and the proposed approach. Section 5 reflects the evaluation results. A comparison of this study and other literature reviews is provided in Section 6. Finally, Section 7 encapsulates the conclusions drawn from the research.

## 2. Multicarrier Modulation Schemes Based on the VLC System

### 2.1. DCO-OFDM

**Figure 1:**
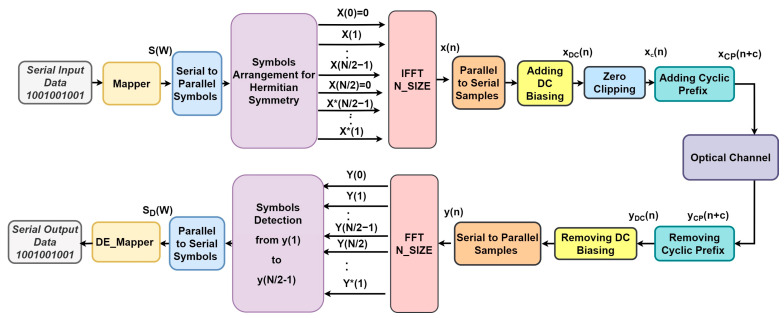
DCO-OFDM block diagram based on a VLC system.

In this depicted framework, located in Figure 1, the initial step at the transmitter involves the mapping of input serial data bits into complex modulated symbols utilizing either M-PSK or M-QAM mapper formats, denoted as S(W), where W ranges from 1 to $\frac{N}{2}-1$. They subsequently convert from a serial to a parallel configuration of length (*N/2-1*) before being applied to the data organization block [22]. The organization block aims to arrange the input complex symbols into the Hermitian symmetry (H.S) format known as X(k), as detailed in (1). The formatted X(k) is then applied to the IFFT stage, where ′*k*′ represents the subcarrier indices.

(1) $$X\left(k\right)=X^{*}\left(N-k\right),\;\;\;1\leq k\leq N/2-1,\;\mathrm{where}\;X\left(0\right)=X\left(N/2\right)=0,$$

In this context, ′*k*′ signifies the size of IFFT, and (.)* denotes the complex conjugate operator. Two different domains for notation exist in the context of the O-OFDM technique: the frequency domain (represented by capital indicators such as *X*(*k*)) and the time domain (represented by lowercase indicators such as *x*(*n*)). Subsequently, the output samples x(n) undergo serialization. In this framework, x(n) is first elevated using a DC-biasing shift, and then the real bipolar data samples are clipped to create unipolar samples, which are designated as $x_{DC}\left(n\right)$. The related signal’s root mean square value and the DC shift, $F_{DC}$, are exactly proportional, as described in Equation (2) [9,23].

(2) $$F_{DC}=A\sqrt{E\left(x^{2}\left(n\right)\right)},$$

Here, A represents a proportionality constant, and the expectation or mean value of the signal is indicated by the symbol E(.). The proportionality constant (A) is calculated as shown in the equation $\gamma_{DC}=10*\log_{10}\left(A^{2}+1\right)\mathrm{dB}$, where $\gamma_{DC}$ is an arbitrary constant. Following the addition of the DC level, denoted as $F_{DC}$, the final represented DC signal is denoted as $x_{DC}\left(n\right)$ [24].

(3) $$x_{DC}\left(n\right)=x\left(n\right)+F_{DC},$$

Subsequently, a clipping operation can occur, leading to the introduction of a clipping noise component denoted as $cn_{DC}$, so

(4) $$x_{c}\left(n\right)=x_{DC}\left(n\right)+cn_{DC},$$

(5) $$x_{c}\left(n\right)=x\left(n\right)+F_{DC}+cn_{DC},$$

Once the signal is clipped to obtain $x_{c}\left(n\right)$, a CP can be appended before the broadcast through the channel. On the receiving side, an inverse operation is performed. Various biasing levels, such as 7, 10, and 13 dB, can be utilized in the DCO-OFDM system prior to the clipping process. The assessment of the DCO-OFDM system hinges on three key variables: the DC-biasing level utilized for the bipolar signal, the impact of clipping noise on the transmitted signal, and the optical power of the transmitted signal relative to the applied DC shift. A trade-off exists among these factors: when the DC shift is raised, clipped noise diminishes, while it results in an energy-inefficient communication system with excessive power dissipation [25].

When employing high modulation orders, such as 512-QAM, and using low DC-biasing levels, such as 7 and 10 dB, the system’s performance is notably subpar, with a high bit error rate of $10^{-3}$ even for high SNR levels. However, the system performs better when employing the same high modulation order but with a higher DC shift of 13 dB. This improvement is attributable to the reduced impact of clipping noise, resulting in a lower BER of $10^{-4}$ even for lower levels of SNR compared to the scenarios at 7 and 10 dB [13].

### 2.2. ACO-OFDM

**Figure 2:**
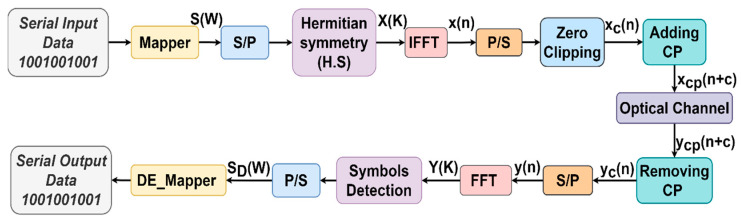
ACO-OFDM block diagram based on a VLC system.

The ACO-OFDM framework with N subcarriers is illustrated in Figure 2. In this system, the input bit stream is transformed into complex symbols, S(W), where W ranges from 1 to $\frac{N}{4}$, based on the chosen mapper scheme, such as M-QAM [26]. H.S is enforced on the OFDM subcarriers to ensure the real-valued nature of the time-domain signals, as dedicated in Equation (1). Specifically, only odd subcarriers are modulated to enable direct clipping of the time-domain signals at zero. The organized symbols for the ACO-OFDM signal are described as follows [27]:

(6) $$X\left(K\right)=\left[0,\;S_{1},\;0,\;S_{2},\ldots ,S_{N/4},0,\;S_{N/4}^{*},\ldots ,S_{2}^{*},\;0,\;S_{1}^{*}\right],$$

By utilizing an IFFT procedure, the time-domain ACO-OFDM signal is generated, as described:

(7) $$x\left(n\right)=\frac{1}{\sqrt{N}}\overset{N-1}{\underset{k=0}{\sum }}X\left(K\right)\;exp\left(j\frac{2\pi }{N}kn\right),\;n=0,1,\ldots ,N-1,$$

Following a symmetrical pattern over half a wave, as:

(8) x(n)=−x(n+N/2),   n=0,1,…,N/2−1,

Therefore, it is possible to clip the negative portion without any corruption in the information.

(9) $$\left\lfloor x_{c}\left(n\right)\right\rfloor =x\left(n\right)+i\left(n\right)=\{\begin{array}{c} x\left(n\right),\;\;\;\;\;\;\;\;\;\;\;\;\;x_{ACO,n}\geq 0;\\ 0,\;\;\;\;\;\;\;\;\;\;\;\;\;\;\;\;x_{ACO,n}\leq 0; \end{array}\;\;\;\;\;\;\;\;\;\;$$

For n=0,1,…,N−1, i(n) indicates ACO-OFDM negative clip distortion.

Following the IFFT operation, the signal is serialized. Subsequently, zero-clipping is applied, and a CP is added at the onset of each OFDM symbol to eradicate ISI at the receiver. This final signal is then utilized to modulate the LEDs [2].

After analyzing the frequency domain at the receiver, it was observed that the negative clipping noise only affected the even subcarriers. Consequently, after removing the CP, the signal is parallelized, and the transmitted signal on the odd subcarriers is readily retrieved at the receiver by a simple FFT process [28].

### 2.3. Flip-OFDM 

**Figure 3:**
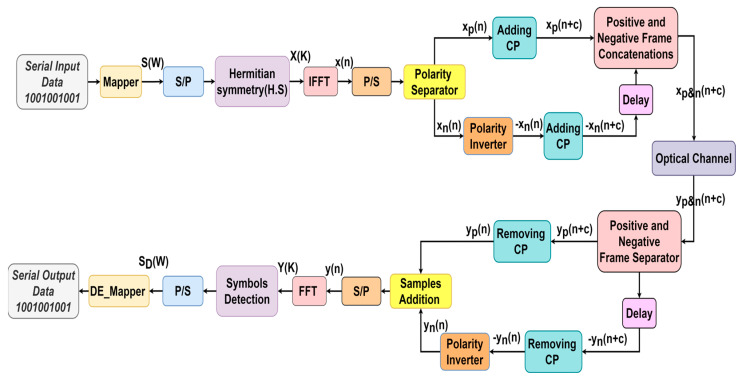
Flip-OFDM block diagram based on a VLC system.

In Figure 3, a schematic of a Flip-OFDM is depicted. The data input process in Flip-OFDM follows a pattern similar to that discussed in DCO-OFDM, extending up to the output generated by the IFFT stage. Flip-OFDM utilizes half of the available OFDM subcarriers for data transmission, leading to a real bipolar output x(n) from the IFFT process [29]. The signal x(n) is divided into two separate components: one comprises the positive samples, denoted as $x_{p}\left(n\right)$, and the other comprises the negative samples, denoted as $x_{n}\left(n\right)$, as specified in Equation (10).

(10) $$x_{p}\left(n\right)=\{\begin{array}{c} x\left(n\right),\;\;\;\;\;\;\;\;\;\;if\;x\left(n\right)\geq 0\\ 0\;,\;\;\;\;\;\;\;\;\;\;\;otherwise \end{array}\;\mathrm{and}\;x_{n}\left(n\right)=\{\begin{array}{c} x\left(n\right),\;\;\;\;\;\;\;\;\;\;if\;x\left(n\right)<0\\ 0\;,\;\;\;\;\;\;\;\;\;\;\;otherwise \end{array}$$

Here, n ranges from 0 to N−1. The $x_{p}\left(n\right)$, is sent within the first OFDM segment, whereas the second OFDM segment is allocated for conveying the flipped $x_{n}\left(n\right)$, as visualized in Figure 3. The first and second OFDM segments have CPs appended to them, with a duration of C, since communication occurs across a dispersive optical channel [30]. Subsequently, the second segment is temporally delayed by (N + C) and synchronized after the first segment. At the Flip-OFDM receiver, the bipolar OFDM frame is reconstructed using the two received segments, as demonstrated in Figure 3. Initially, the CPs associated with each segment are released, facilitating the regeneration of the original bipolar samples as follows:

(11) $$y\left(n\right)=y_{p}\left(n\right)+y_{n}\left(n\right)$$

Here, $y_{p}\left(n\right)$ represents the samples of the first received segment, and $y_{n}\left(n\right)$ represents the samples of the second segment [22].

### 2.4. ADO-OFDM

**Figure 4:**
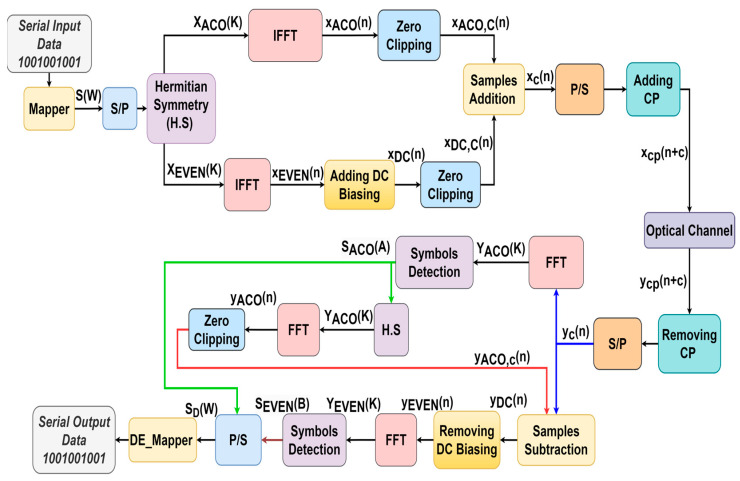
ADO-OFDM block diagram based on a VLC system.

A hybrid technique combines data arrangement between even and odd subcarriers, known as ADO-OFDM. It operates similarly to ACO-OFDM by utilizing the odd subcarriers, but it also incorporates even subcarriers, resembling DCO-OFDM [31]. Figure 4 provides an overview of the block diagram of ADO-OFDM. As mentioned earlier regarding the disadvantages of ACO and DCO-OFDM, ADO-OFDM is employed to enhance the performance of these OFDM schemes. It involves mapping the symbols of S(W), where W ranges from 1 to N/2 − 1. S(W) is applied to H.S as denoted as X(K). X(K) is divided into two parts as dedicated in Equation (13) for odd subcarriers and Equation (14) for even subcarriers [31,32,33].

(12) $$X\left(K\right)=\left[0,\;S_{1},\;S_{2},\ldots \ldots ,S_{N/2-1},0,\;S_{N/2-1}^{*},\ldots \ldots ,S_{2}^{*},\;S_{1}^{*}\right],$$

(13) $$X_{ACO}\left(K\right)=\left[0,\;S_{1},\;0,\;S_{4},0,\ldots \ldots ,0,S_{N/4},0,\;S_{N/4}^{*},0,\ldots \ldots ,S_{2}^{*},0,\;S_{1}^{*}\right],$$

(14) $$X_{EVEN}\left(K\right)=\left[0,\;0,S_{\frac{N}{4}+1},\;0,\;S_{\frac{N}{4}+2},0,\ldots \ldots ,0,S_{\frac{N}{2}-1},0,\;0,\;0,\;S_{\frac{N}{2}-1}^{*},0,\ldots \ldots ,S_{\frac{N}{4}+2}^{*},0,\;S_{\frac{N}{4}+1}^{*},0\right],$$

Both $X_{ACO}\left(K\right)$ and $X_{EVEN}\left(K\right)$ components are applied to IFFT operation as follows:

(15) $$x_{ACO}\left(n\right)=\frac{1}{\sqrt{N}}\overset{N-1}{\underset{k=0}{\sum }}X_{ACO}\left(k\right)\;exp\left(j\frac{2\pi }{N}kn\right),\;\;\;n=0,1,\ldots ,N-1$$

(16) $$x_{EVEN}\left(n\right)=\frac{1}{\sqrt{N}}\overset{N-1}{\underset{k=0}{\sum }}X_{EVEN}\left(k\right)\;exp\left(j\frac{2\pi }{N}kn\right),\;\;\;n=0,1,\ldots ,N-1$$

### 2.5. ASCO-OFDM

**Figure 5:**
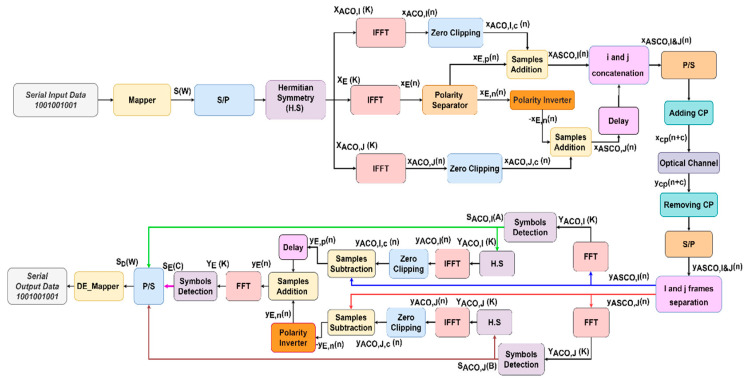
ASCO-OFDM block diagram based on a VLC system.

Figure 5 depicts the framework of the ASCO-OFDM scheme. It involves mapping S(W) symbols, where W is the interval from 1 to $\frac{3N}{4}-1$. To ensure the generation of a real signal, it is necessary to establish H.S for S(W) symbols. The H.S signal is divided into $X_{ACO,I}\;\left(K\right)$,$\;X_{ACO,J}\left(K\right)$, and $X_{E}\left(K\right)$ prior to the IFFT operation. $X_{ACO,I}\;\left(K\right)$ and $X_{\mathrm{ACO},J}\left(K\right)$ with size N consisting of $\frac{N}{4}$ complex symbol (CS) and their complex conjugate (CC), located only in the odd subcarriers, are integrated with zero values into the even subcarriers. $X_{E}\left(K\right)$ with size *N* consisting of $\frac{N}{4}-1$ CS and their CC, located in the even subcarriers, are integrated with zero values into the odd subcarriers. $X_{ACO,I}\;\left(K\right)$, $X_{ACO,J}\left(K\right)$ and $X_{E}\left(K\right)$ are as designated in Equations (17)–(19), respectively. They undergo processing using an IFFT process to generate real bipolar $x_{ACO,I}\left(n\right)$, $x_{ACO,j}\left(n\right)$, and $x_{E}\left(n\right)$ signals, respectively [22]:

(17) $$X_{ACO,I}\;\left(K\right)=\left[0,\;S_{1},\;0,\;S_{2},\;\ldots \ldots ,\;0,\;S_{N/4},\;0,\;S_{N/4}^{*},\;0,\ldots \ldots ,\;S_{2}^{*},\;0,\;S_{1}^{*}\right],$$

(18) $$X_{ACO,J}\left(K\right)=\left[0,\;S_{\frac{N}{4}+1},\;0,\;S_{\frac{N}{4}+2},\;\ldots \ldots ,\;0,\;S_{N/2},\;0,\;S_{N/2}^{*},\;0,\ldots \ldots ,\;S_{\frac{N}{4}+2}^{*},\;0,\;S_{\frac{N}{4}+1}^{*}\right],$$

(19) $$\;X_{E}\left(K\right)=\left[0,\;0,\;S_{\frac{N}{2}+1},\;0,\;S_{\frac{N}{2}+2},\;\ldots \ldots ,\;S_{\frac{3N}{4}-1},\;0,\;0,\;0,\;S_{\frac{3N}{4}-1}^{*},\ldots \ldots ,\;S_{\frac{3N}{4}+2}^{*},\;0,\;S_{\frac{3N}{4}+1}^{*},\;0\right],$$

To fulfill the non-negative signal requirement, any negative values within $x_{ACO,I}\left(n\right)$ and $x_{ACO,j}\left(n\right)$ are clipped to zero, yielding $x_{ACO,I,C}\left(n\right)$, and $x_{ACO,J,C}\left(n\right)$ as described below [21]:

(20) $$x_{ACO,I,C}\left(n\right)=0.5\left(x_{ACO,I}\left(n\right)+\left|x_{ACO,I}\left(n\right)\right|\right),$$

(21) $$x_{ACO,J,C}\left(n\right)=0.5\left(x_{ACO,I}\left(n\right)+\left|x_{ACO,I}\left(n\right)\right|\right),$$

When eliminating the negative samples in $x_{E}\left(n\right)$ through clipping, half of the information originally carried in $x_{even}\left(n\right)$ is forfeited, as there is no relationship between $x_{E}\left(n\right)\;\mathrm{and}\;x_{E}\left(n+N/2\right)$. Consequently, $x_{E}\left(n\right)$ divides into $x_{E,p}\left(n\right)$ and $x_{E,n}\left(n\right)$. $x_{E,p}\left(n\right)$ and $x_{E,n}\left(n\right)$ are formed to transmit the bipolar samples of $x_{E}\left(n\right)$. Specifically,$\;x_{E,p}\left(n\right)$ represents the scenario where all the negative values within $x_{E}\left(n\right)$ are clipped to zero. $x_{E,n}\left(n\right)$ depicts the case where all the positive values in $x_{E}\left(n\right)$ are clipped to zero, and the remaining negative values are transformed into positive values. They can be expressed as follows [18]:

(22) $$x_{E,p}\left(n\right)=0.5\left(x_{E}\left(n\right)+\left|x_{E}\left(n\right)\right|\right),$$

(23) $$x_{E,n}\left(n\right)=0.5\left(-x_{E}\left(n\right)+\left|x_{E}\left(n\right)\right|\right),$$

Therefore, ASCO-OFDM uses two consecutive segments for data transmission, denoted as $x_{ASCO,I}\left(n\right)$ and $x_{ASCO,J}\left(n\right)$, and they can be expressed as follows:

(24) $$x_{ASCO,I}\left(n\right)=x_{ACO,I,C}\left(n\right)+x_{E,p}\left(n\right)=0.5\left(x_{ACO,I}\left(n\right)+\left|x_{ACO,I}\left(n\right)\right|+x_{E}\left(n\right)+\left|x_{E}\left(n\right)\right|\right),$$

(25) $$x_{ASCO,J}\left(n\right)=x_{ACO,J,C}\left(n\right)+x_{E,n}\left(n\right)=0.5\left(x_{ACO,J}\left(n\right)+\left|x_{ACO,J}\left(n\right)\right|-x_{E}\left(n\right)+\left|x_{E}\left(n\right)\right|\right),$$

The transmitted signals, $x_{ASCO,I}\left(n\right)$ and $x_{ASCO,J}\left(n\right)$, are concatenated as denoted $x_{ASCO,I\&j}\left(n\right)$, equipped with cyclic prefixes, represented as $x_{cp}\left(n+c\right)$. This signal is transmitted via LED. Upon reception, they undergo processing through an inverse procedure, mirroring the operation carried out at the transmitter [18].

### 2.6. LACO-OFDM

**Figure 6:**
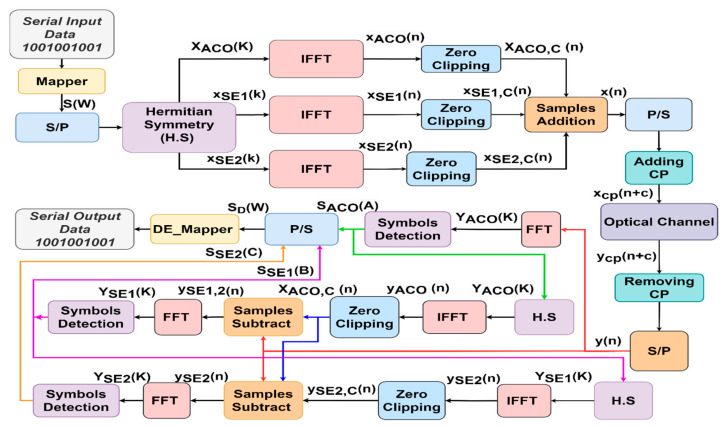
Three-layered LACO-OFDM block diagram based on a VLC system.

LACO-OFDM’s three-layer structure is depicted in Figure 6. The input signal S(W) was divided into the necessary number of layers after undergoing the H.S operation to achieve a predetermined output data rate. The overall composite signal was created by merging frames, each consisting of a defined number of symbols, each with a size of N. The signalling strategy in the first layer (L=1) closely emulated that of ACO-OFDM, as elucidated in references [19,34,35]. This layer included N/4 CS, with their CC allocated in the odd subcarriers and integrated with zero in the even subcarriers, as represented in Equation (26).

(26) $$X_{ACO}\;\left(K\right)=\left[0,\;S_{1},\;0,\;S_{2},\;\ldots \ldots ,\;0,\;S_{N/4},\;0,\;S_{N/4}^{*},\;0,\ldots \ldots ,\;S_{2}^{*},\;0,\;S_{1}^{*}\right],$$

In L=2,  N/8 CS with their CC are allocated to the even subcarriers and integrated with zero in the other subcarriers, as depicted in Equation (27).

(27) $$X_{SE,1}\;\left(K\right)=\left[0,0,\;S_{\frac{N}{4}+1},\;0,0,0,\;S_{\frac{N}{4}+2},\;\ldots \ldots ,\;0,\;S_{\frac{3N}{8}},\;0,0,0,\;S_{\frac{3N}{8}}^{*},\;0,\ldots \ldots ,\;S_{\frac{N}{4}+2}^{*},\;0,0,0,\;S_{\frac{N}{4}+1}^{*},0\right],$$

In *L* = *3*, *N*/*16* CS with their CC are assigned to the even subcarriers and zeros integrated into the residual subcarriers, as depicted in Equation (28).

(28) $$X_{SE,2}\;\left(K\right)=\left[0,0,0,0,\;S_{\frac{3N}{8}+1},\;0,0,0,0,0,0,0,\;S_{\frac{3N}{8}+2},\;\ldots \ldots ,\;0,\;S_{\frac{7N}{16}},\;0,0,0,0,0,0,0,\;S_{\frac{7N}{16}}^{*},\;0,\ldots \ldots ,\;S_{\frac{3N}{8}+2}^{*},\;0,0,0,\;0,0,0,0,S_{\frac{3N}{8}+1}^{*},0,0,0\right],$$

Generally, for L>1, $\left(N/2^{L+1}\right)$ CC and their CS are transmitted on subcarriers $2^{L-1}\left(2m+1\right)$, where *m* ranges from 0 to $N/2^{L+1}-1$. Subsequently, the residual subcarriers are set to zero. $X_{ACO}\left(K\right)$,$\;X_{SE,1}\left(K\right)$, and $X_{SE,2}\left(K\right)$ are transformed into the time domain using three IFFT stages, and the resulting signals are denoted as $x_{ACO}\left(n\right)$, $x_{SE,1}\left(n\right)$, and $x_{SE,2}\left(n\right)$, respectively. The signals $x_{ACO}\left(n\right)$, $x_{SE,1}\left(n\right)$, and $x_{SE,2}\left(n\right)$, after zero clipping, are combined for single-segment transmission before the addition of the CP, as depicted in Figure 6. In this scenario, applying clipping to the IFFT resulting signal of one layer can introduce distortion in the frequency domain for the subsequent layer [35]. 

Therefore, in the data recovery at the receiver, a successive detection approach was employed, as outlined in [35], where symbols were reconstructed layer by layer from the received signal y(n), as illustrated in Figure 6. It is worth noting that for L=1, the modulated symbols remained unaffected by distortion. The associated symbols, $Y_{ACO}\left(k\right)$, can be directly identified, like in ACO-OFDM, as described in Section 2.2. These symbols were subsequently utilized in the second layer to eliminate the corresponding distortion before proceeding with signal detection for the $L^{th}$ layer, following the procedure depicted in Figure 6. As previously explained, LACO, in contrast to ACO, simultaneously transmits different layers, enabling the utilization of varying constellation sizes within each layer to attain a specified data rate. Consequently, it can achieve its target BER with a lower required SNR. In essence, LACO offers improved spectral efficiency and the added benefit of reducing PAPR [19].

## 3. Assessment Criteria Employed to Judge the Effectiveness of MCM Approaches

The evaluation of MCM schemes involves an analysis of factors such as the data arrangement, which directly influences spectral efficiency (SE), in addition to power efficiency, computational complexity, BER, and PAPR [2,36,37,38,39].

### 3.1. Data Arrangement and Spectral Efficiency 

**Figure 7:**
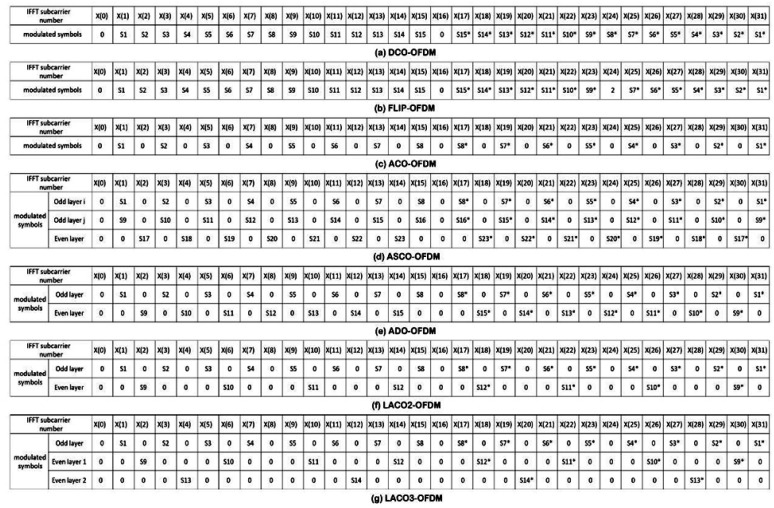
The modulated symbol distribution to the different O-OFDM schemes (for example, 32-IFFT size).

The data arrangement preceding the H.S operation, necessary to yield a real bipolar signal after the IFFT process, varies from one MCM scheme to another, as shown in Figure 7. As a result, multiple MCM schemes, including those mentioned earlier, lead to differing output spectral efficiencies [35,40].

DCO-OFDM employs a single IFFT in the transmitter and a single FFT in the receiver, utilizing a DC bias to transmit signals in a unipolar and real form via an LED. Notably, DCO-OFDM exhibited superior SE compared to other O-OFDM methods employed in VLC. The SE of this scheme is expressed in Table 1 [41]. 

Here, M represents the modulation order, and N denotes the IFFT/FFT size utilized in the analysis. This heightened efficiency can be attributed to the symbol structure before its application to the IFFT, as indicated in the Figure 7. Furthermore, considering the symbol structure, it is evident that the data rate of DCO-OFDM was 50% compared to conventional OFDM used in RF communication systems. The ADO-OFDM symbol structure reveals that the data rate of this method matched that of the DCO-OFDM approach, indicating it as 50% of the data rate employed in conventional OFDM for RF communication systems, as mentioned in Figure 7 and Table 1. Here, $M_{odd}$ represents the modulation order used for the odd subcarriers, and $M_{even}$ represents the modulation order used for the even subcarriers in the ADO-OFDM system. 

ACO-OFDM and FLIP-OFDM had identical SEs. Furthermore, ACO-OFDM’s data rate was 25% of that used in conventional OFDM for RF communication systems and was 50% lower than the data rate of DCO-OFDM, as shown in the Figure 6. Based on the earlier explanation of the symbol arrangement shown in Figure 7, the ASCO-OFDM spectral efficiency evidenced that the data rate of this method was 37.5% in comparison to conventional OFDM used in RF communication systems [42]. The ASCO-OFDM’s SE formula is shown in Table 1. $M_{odd,i}$ denotes the modulation order applied to the odd subcarriers in the first IFFT process, $M_{odd,j}$ represents the M for the odd subcarriers in the second IFFT process, and $M_{even}$ signifies the M for the even subcarriers in the third IFFT process [9].

The data rate of a single-layer LACO-OFDM matched that of ACO-OFDM. However, when we introduced an additional layer to create a two-layer LACO-OFDM, the data rate increased to match that of ASCO-OFDM, as illustrated in Table 1. Within this context, $M_{1}$ represents the modulation order assigned to the odd subcarriers in the first IFFT process, $M_{2}$ designates the modulation order for the specific even subcarriers in the second IFFT process, and N denotes the IFFT size employed in the analysis. With each subsequent increment in the number of layers, the data rate consistently met an increase. Generally, varying signal constellation sizes are employed across different layers, denoted as $M_{l}-QAM$ in the $l^{th}$ layer to adjust the overall SE, the SE of LACO-OFDM can be articulated as follows [19]:

(29) $$\gamma_{LACO,l}=\frac{\sum_{l=1}^{L}\frac{1}{2^{l}}log_{2}\;\left(M_{l}\right)}{1+\frac{2}{N}}\;\;\;\;\;\;\;\;\;\;\;\;\;\;\;\mathrm{bits}/s/\mathrm{Hz},$$

**Table 1:** Spectral efficiency formula for various O-OFDM approaches and the evaluation values (bits/sec/Hz) for various constellation sizes at an FFT/IFFT size of 1024 [2,43].

MCM TECHNIQUE	Spectral Efficiency Formula	4-QAM	16-QAM	64-QAM	256-QAM	1024-QAM
DCO-OFDM	$\frac{{log}_{2}M}{1+\frac{2}{N}}$	1.9961	3.9922	5.9883	7.9844	9.9805
ACO-OFDM	$\frac{\frac{{log}_{2}M}{2}}{1+\frac{2}{N}}$	0.9981	1.9961	2.9942	3.9922	4.9903
FLIP-OFDM	$\frac{\frac{{log}_{2}M}{2}}{1+\frac{2}{N}}$	0.9981	1.9961	2.9942	3.9922	4.9903
ASCO-OFDM	$\frac{\frac{{log}_{2}M_{odd,i}}{4}+\frac{{log}_{2}M_{odd,j}}{4}+\frac{{log}_{2}M_{even}}{4}}{1+\frac{2}{N}}$	1.4971	2.9942	4.4912	5.9883	7.4854
ADO-OFDM	$\frac{\frac{{log}_{2}M_{odd}}{2}+\frac{{log}_{2}M_{even}}{2}}{1+\frac{2}{N}}$	1.9961	3.9922	5.9883	7.9844	9.9805
LACO_2-OFDM	$\frac{\frac{{log}_{2}M_{1}}{2}+\frac{{log}_{2}M_{2}}{4}}{1+\frac{2}{N}}$	1.4971	2.9942	4.4912	5.9883	7.4854
LACO_3-OFDM	$\frac{\frac{{log}_{2}M_{1}}{2}+\frac{{log}_{2}M_{2}}{4}+\frac{{log}_{2}M_{3}}{8}}{1+\frac{2}{N}}$	1.7466	3.4932	5.2398	6.9864	8.7329
LACO_4-OFDM	$\frac{\frac{{log}_{2}M_{1}}{2}+\frac{{log}_{2}M_{2}}{4}+\frac{{log}_{2}M_{3}}{8}+\frac{{log}_{2}M_{4}}{16}}{1+\frac{2}{N}}$	1.8713	3.7427	5.6140	7.4854	9.3567

**Figure 8:**
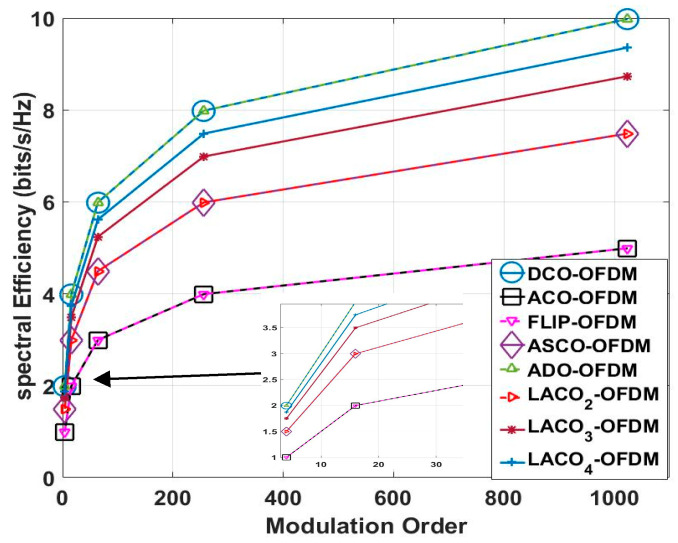
The fluctuations in spectral efficiency regarding changes in modulation order were examined while maintaining a fixed FFT/IFFT size of 1024 for various O-OFDM schemes.

As a conclusion, Table 1 and Figure 8 display the variations in SE as the M changes, with the FFT/IFFT size fixed at 1024 for the previously mentioned schemes. In this study, the authors observed that as the M increased, the SE also increased. Notably, the SE of DCO-OFDM and ADO-OFDM at 4-QAM matched the SE of ACO-OFDM and FLIP-OFDM at 16-QAM. Furthermore, the SE of ACO-OFDM and FLIP-OFDM at 64-QAM equalled the SE of ASCO-OFDM and two-layer LACO-OFDM at 16-QAM.

**Figure 9:**
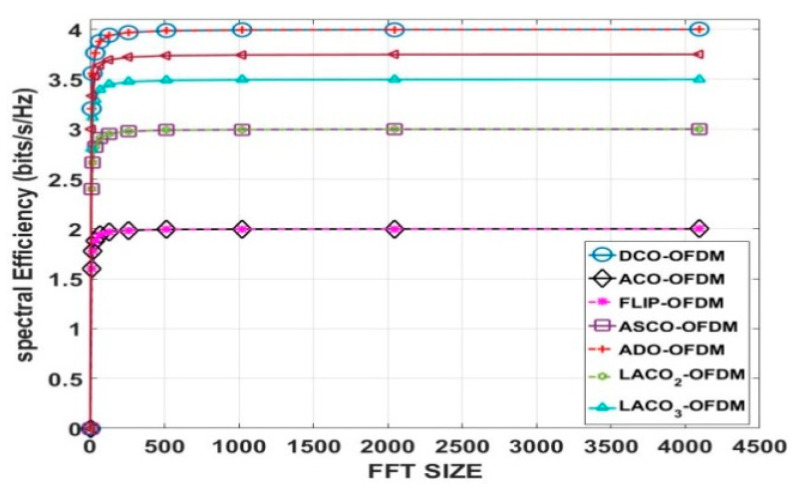
The fluctuations in spectral efficiency are examined concerning changes in FFT/IFFT size while maintaining a modulation scheme of 16-QAM for various O-OFDM schemes.

In particular, Figure 9 and Table 2 illustrate the SE of the previously mentioned methods at M equaling 16-QAM. This figure illustrates that as the FFT/IFFT size increases, there comes a point where the SE does not exhibit a significant improvement.

### 3.2. Power Efficiency

As previously stated, the data rate depends on the arrangement of symbols in each approach, with the H.S principle being a necessary condition to generate real bipolar samples following the IFFT operation. The conversion from bipolar to unipolar involves two methods. First, a DC bias is added to the bipolar signal, followed by clipping it to zero, as seen in DCO-OFDM and even part of ADO-OFDM. Consequently, DCO and ADO-OFDM are considered power-inefficient O-OFDM techniques. The amount of additional DC bias controls how well these techniques work [13,40].

Second, various schemes clipped the real output bipolar samples to zero, as employed in ACO-OFDM, FLIP-OFDM, ASCO-OFDM, and LACO-OFDMD. The utilization of zero-clipping operations, made possible by the symbol H.S structure of these techniques, enhances symbol detection in the receiver, resulting in superior performance compared to DCO-OFDM and ADO-OFDM. Ultimately, FLIP, ACO, LACO, and ASCO-OFDM stand out as power-efficient OFDM techniques [2,19,44].

### 3.3. Computational Complexity

The concept of computational complexity relates to the number of operations needed for both the IFFT/FTT at the transmitter and receiver [45,46]. Table 3 presents the computational complexity of the previously mentioned approaches [9,22]. The ACO, Flip, and DCO-OFDM computational complexity is $O(Nlog_{2}N)$ for both the transmitter and receiver. For ADO and two-layer LACO-OFDM, the transmitter complexity is $2O(Nlog_{2}N)$, while the receiver complexity is $3O(Nlog_{2}N)$. In the case of ASCO-OFDM and three-layer LACO-OFDM, the transmitter complexity is $3O(Nlog_{2}N)$, and the receiver complexity is $5O(Nlog_{2}N)$.

The FFT operation typically exhibits a complexity of $O(Nlog_{2}N)$ when using a standard Cooley–Tukey radix-2 FFT algorithm [22]. The IFFT operation is quite similar to the FFT, differing mainly in scaling factors, and it also has a complexity of $O(Nlog_{2}N)$. The big O notation is a mathematical tool used to classify the limiting behavior of a function as its running time or resource usage scales with the input size. The number of multiplications and additions needed to compute the FFT/IFFT transform is often referred to as the computational hardware complexity. The quantities of multiplications $M_{FFT/IFFT}$ and additions $S_{FFT/IFFT}$ required to compute the real-valued FFT can be expressed as follows. However, the actual values for $M_{FFT/IFFT}$ and $S_{FFT/IFFT}$ would depend on the specific FFT/IFFT implementation and the size of the input data [9,47].

(30) $$M_{FFT/IFFT}=N*{log}_{2}N-3N+4,$$

(31) $$S_{FFT/IFFT}=3N*{log}_{2}N-3N+4,$$

**Figure 10:**
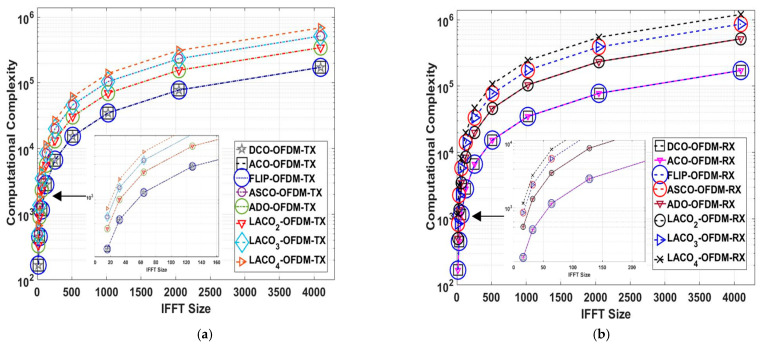

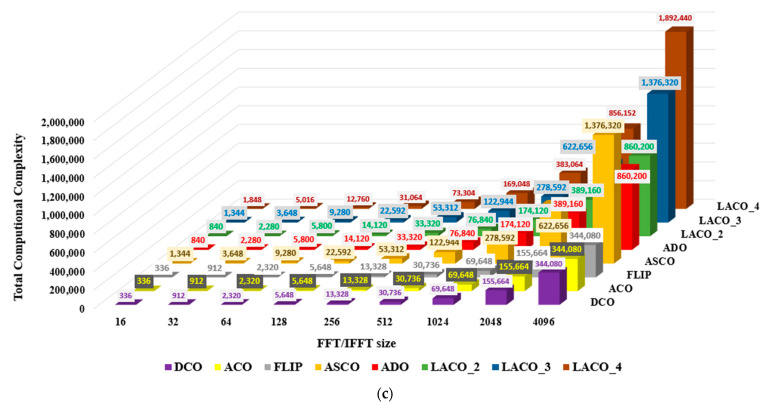
The computational complexities across different O-OFDM approaches with varying FFT/IFFT sizes at both the transmitter and receiver: (**a**) transmitter, (**b**) receiver, and (**c**) total computational complexity for the different approaches.

These equations illustrate that the number of $M_{FFT/IFFT}$ and $S_{FFT/IFFT}$ for the FFT operations grow logarithmically with the input data size, which is a key characteristic of FFT’s efficiency in handling large datasets. As the size of the FFT/IFFT increases, the computational complexity increases, as illustrated in Figure 10. Notably, this increase in computational demand aligns with an improvement in spectral efficiency, as depicted in Figure 8. As a result, the authors opted to use a 1024 FFT/IFFT size in their simulation analysis, as it represents an optimal choice that strikes a balance between obtaining high SE and minimizing computational complexity [43,47]. 

Table 4 provides the number of the number of $M_{FFT/IFFT}$ and $S_{FFT/IFFT}$ operations for the mentioned schemes when the IFFT/FFT size is 1024. This table should clearly summarise the computational complexities of these techniques for this specific input size. 

### 3.4. Bit Error Rate (BER)

BER is an essential metric for evaluating the performance of a data transmission system. It gauges the occurrence rate of errors during data transmission, providing valuable insights into the probability of bit errors within a specific data stream. The exact definition of BER is the ratio of incorrect bits to total bits transmitted. This ratio is a fundamental indicator of the quality and reliability of a communication link [2]. A lower BER reflects greater accuracy and dependability in data transmission, underscoring the strength and reliability of the communication channel. The formula for calculating the BER is straightforward and can be expressed as follows:

(32) $$BER=\frac{Number\;of\;Erorr\;Bits}{Total\;Number\;of\;Transmitted\;Bits}$$

While BER remains a critical parameter, communication transmission systems often incorporate additional metrics, including SNR and energy per bit to noise power spectral density ratio $E_{b}/N_{O}$. SNR measures the power of the desired signal in relation to the background noise, while $E_{b}/N_{O}$ represents the ratio of energy per bit to the noise power spectral density. These parameters are closely tied to the performance and quality of the transmitted signal and are commonly used alongside BER to perform a thorough evaluation of the system. It is important to acknowledge that each distinct modulation scheme possesses its unique error characteristic. This disparity arises from the varying performance of modulation types when exposed to noise. Specifically, higher modulation schemes (such as 64-QAM and others) capable of transmitting higher data rates tend to be less resilient in noisy environments. Conversely, lower modulation formats (like BPSK and QPSK) provide lower data rates but exhibit higher resistance to the effects of noise. The subsequent simulation results section outlines the BER performance of the previously mentioned systems.

### 3.5. Peak-to-Average Power Ratio (PAPR)

PAPR, in wireless communication O-OFDM systems, is a metric that quantifies the ratio between the signal peak and its average power. High PAPR presents a significant challenge in O-OFDM systems, leading to heightened non-linearity and unwanted deformations. The mathematical relationship for PAPR can be described as follows [48].

(33) $$PAPR=\frac{max\left\{{\left|{\left\lfloor a_{OOFDM,n}\right\rfloor }_{c}\right|}^{2}\right\}}{E{|{\left\lfloor a_{OOFDM,n}\right\rfloor }_{c}|}^{2}},$$

In this context, E{.} represents the mathematical expectation, while ${\left\lfloor a_{OOFDM,n}\right\rfloor }_{c}$ signifies the resulting output time-domain samples from the IFFT operation after applying the previously discussed clipping process in various O-OFDM approaches [24].

In this paper, the authors present an innovative approach to address the PAPR challenge in the previously mentioned O-OFDM approaches. Typically, the effectiveness of this PAPR reduction methodology is evaluated by utilizing the complementary cumulative distribution function (CCDF). The CCDF quantifies the probability that the PAPR of an O-OFDM signal exceeds a specified threshold PAPR0, as described below [3]:

(34) $$CCDF=P\left(PAPR>PAPR0\right)=1-{\left(1-e^{-PAPR0}\right)}^{N},$$

If a particular scheme outperforms all others in terms of PAPR methodology, its CCDF curve will be positioned to the left of the others. Therefore, in the simulation results section, we analyze and discuss the juxtaposition between the CCDF curves of the proposed schemes.

## 4. PAPR Reduction Methodology and Proposed Model

This section provides a comprehensive exploration of the PAPR reduction technique and outlines the proposed model methodology employed for implementing this reduction technique within the context of the various O-OFDM configurations discussed earlier.

To some extent, the conventional PAPR reduction methods may apply to both traditional OFDM and O-OFDM systems. Still, there are specific considerations and challenges unique to O-OFDM that warrant clarification. The transmitted signal of conventional OFDM is complex and bipolar, but in O-OFDM, the transmitted signal must be real and unipolar. The simulation results in Section 5.1 illustrate the change in BER performance of the bipolar DCO-OFDM signal before DC-biasing and zero clipping. With the unipolar DCO-OFDM signal with different DC-biasing, the performance after DC-biasing and zero clipping is decreased. The same behaviour is achieved in Section 5.2 for the ACO-OFDM. This illustrates the PAPR reduction methods that can be used in both OFDM systems, but with different PAPR and BER performance evaluations. However, sometimes, we must modify the PAPR reduction technique to meet the VLC transmission requirement while operating at different reduction parameter values.

To the best of the authors’ knowledge, we can categorize the PAPR mitigation approaches for MCM techniques as adding signal techniques (AST), multiple signal representation (MSR) techniques, and coding techniques (CT)/precoding techniques (PCT) [49].

AST reduces PAPR through three primary techniques: signal clipping, the compression of large peaks using non-linear companding transform (NCT), and the application of peak reduction signals/stretching the constellation. These methods introduce distortion noises into the broadcast signal that cannot be eliminated. Among these techniques, NCT stands as the most widely used and promising one. It involves adding a companding function to the initial transmitted signal, enhancing small signal amplitudes, and compressing high signal amplitudes. The companding function maintains the signal’s average power constant. Various types of NCT include the A-law and μ-law techniques [24]. These two techniques can be easily used for conventional OFDM without any modification for O-OFDM systems but at different companding values to achieve better performance in both PAPR and BER.

MSR approaches generate alternative signals from the same source signal by altering phases, amplitudes, or data positions. The two prevalent types of MSR techniques are partial transmit sequence (PTS) and selective mapping (SLM) [50]. Yet, this approach carries substantial downsides, like increased computational complexity and the need for extra data transmission to the receiver, which reduces overall bandwidth efficiency.

VLC prioritizes lower computational complexity, which is crucial for real-world applications. However, VLC with OOFDM exhibits lower spectral efficiency than conventional OFDM. Consequently, MSR is not the favoured option for mitigating PAPR in VLC systems.

CT can lead to increased side information, computational complexity, or may only function with a limited number of subcarriers, making it inefficient for high-speed VLC-OOFDM communication. To address these drawbacks, PCT emerges as a solution. PCT techniques can be easily used for both conventional and optical OFDM. These techniques use orthogonal matrix multiplication with the complex modulated symbols output from the mapper process. The output signal from this multiplication is applied to the IFFT process in conventional, but in the optical OFDM, it is applied to the Hermitian symmetry and then applied to the IFFT process. The inverse orthogonal matrix is applied to the receiver side before the received signal is applied to the de-mapper process.

We found that the precoding techniques effectively minimize PAPR without compromising BER efficiency, requiring no additional side information or extra processing unlike various precoding methods like Discrete Cosine Transform (DCT), Discrete Hartley Transform (DHT), Walsh-Hadamard Transform (WHT), Vandermonde-Like Matrix transform (VLM), Zadoff–Chu Matrix Transform (ZCT), and precoding with Discrete Fourier Transform (DFT).

Furthermore, the VLM approach demonstrated the highest reduction in PAPR compared to other methods. Therefore, we applied the VLM precoding technique to the various OOFDM approaches to mitigate high PAPR issues.

### 4.1. PAPR Reduction Methodology

In this study, we introduced and explained VLM methodology as a precoding technique (PCT) [50]. This technique is employed to tackle the challenge of high PAPR in communication schemes, focusing on VLC systems using LED transmitters. The primary objective of VLM is to reconfigure the power distribution of the transmitted signal across both the frequency and time domains, aiming to minimise signal peaks and achieve a more uniform power distribution [3].

The VLM methodology achieves this power redistribution by utilizing signal processing algorithms, including precoding matrices of dimensions N×N. This matrix is applied before the IFFT stage to alter the transmitted signal before sending it through the LED. These techniques carefully manipulate the signal characteristics to reduce PAPR and optimize power distribution [49].

(35) $$pc=\left[\begin{array}{ccc} p_{00} & p_{01}\ldots \ldots \ldots \ldots \ldots . & p_{0\left(n-1\right)}\\ \begin{array}{c} p_{10}\\ \vdots \end{array} & \begin{array}{c} p_{11}\ldots \ldots \ldots \ldots \ldots .\\ \vdots \end{array} & \begin{array}{c} p_{1\left(n-1\right)}\\ \vdots \end{array}\\ p_{\left(n-1\right)0} & p_{\left(n-1\right)1}\ldots \ldots \ldots \ldots . & p_{\left(n-1\right)\left(n-1\right)} \end{array}\right],$$

Here, ‘pc’ represents a precoding matrix with dimensions N×N. By redistributing power, the transmitted signal is guaranteed to stay within the LEDs’ linear functioning range via the VLM methodology, avoiding the nonlinear region where distortion is more likely to occur. This approach enhances the overall signal quality and efficiently utilises the LED’s power characteristics.

The VLM technique plays a crucial role in preventing distortion and inefficiency in LED transmission by mitigating the presence of large signal peaks. This is of paramount importance because LEDs possess a limited dynamic range and may exhibit nonlinear behavior when operated near their maximum power levels. The reduction of significant signal peaks helps prevent nonlinear distortion and safeguards system performance [20].

Through the adoption of the VLM method, VLC systems can effectively alleviate the negative consequences of high PAPR, including nonlinear distortion and inefficient LED transmission. This, in turn, leads to enhanced system performance, increased reliability, and improved spectral efficiency in VLC communication [51,52].

The VLM precoding technique is a newly utilized method in O-OFDM systems. It is employed to reduce the PAPR of the transmitted signal. By reducing the autocorrelation between the data, the VLM precoding technique helps mitigate the occurrence of high-power peaks in the transmitted signal. This contributes to a more efficient utilization of the power amplifiers and reduces the likelihood of distortion and nonlinear effects.

Within the present study, the specific VLM matrix utilized is described in Equation (36). The structure and composition of this VLM matrix are specifically designed to achieve effective PAPR reduction in the mentioned O-OFDM systems [50].

(36) $$PC_{mn}=\sqrt{\frac{2}{N+1}}\cos\left(\frac{2}{N+1}\left(m-1\right)\left(n-1\right)\right)\;,$$

where 0≤m,n≤N−1, the $m^{\mathrm{th}}$ row index, $n^{th}$ column index, and N represents the matrix size.

The VLM precoding matrix is invertible. This means that using the inverse operation of this transformation, the original signal may be precisely recovered at the receiver side. The invertibility of this transform is a crucial property that allows for reliable signal reconstruction and decoding. It ensures that the information contained in the transformed domain may be reliably decoded and reconstructed in its original form.

### 4.2. Proposed PAPR Reduction System

This section offers insight into the algorithms employed for various O-OFDM approaches within VLC communication, as illustrated in Algorithms 1 and 2. The most effective issue that causes the system’s performance drawbacks is a high PAPR. By introducing the VLM precoding method for high PAPR mitigation, we aimed to provide insights into their potential benefits in VLC systems. As the authors know, this is the first time the VLM precoding technique has been used for the FLIP, ADO, and LACO-OFDM techniques. 

We acknowledge that precoding techniques for PAPR reduction have been explored in the context of OFDM and ACO-OFDM systems. However, we want to emphasize our manuscript’s unique contributions and innovations introduced by Algorithm 1.

Algorithm 1 presents the emulation procedures for the transmitter side, which can be used for the previously mentioned approaches to mitigating the drawbacks of the high PAPR issue, while Algorithm 2 is employed to emulate receiver procedures. 

The effectiveness of Algorithm 1 lies in its ability to achieve significant PAPR reduction while maintaining robust performance across different VLC techniques. Our experimental results demonstrate the superior performance of the proposed technique compared to existing PAPR results for the conventional MCM approaches, particularly in terms of bit error rate (BER) performance and the absence of side information requirements.

Furthermore, the novelty of Algorithm 1 lies in its adaptability and versatility, enabling seamless integration with various VLC techniques without compromising performance or complexity. By providing insights into the design rationale and performance evaluation of Algorithm 1, our paper contributes to advancing the state-of-the-art in PAPR reduction for the FLIP, DCO, ADO, ACO, LACO, and ASCO-OFDM techniques in VLC communication systems. Section 6 illustrates a comparison between the results achieved and results from related studies.
**Algorithm 1**. Transmitter emulation procedures.1. $K=\log_{2}M$; ← Number of bits per symbol and M is constellation order2. Z, N; ← Number of OOFDM symbols and Number of subcarrier3. if OOFDM is DCO or OOFDM is FLIP or OOFDM is ADO  **then**4.       $symbols_{frame}=N/2$; Number of modulated symbol per OOFDM frame 5.    **Else**
6.       if OOFDM is ACO **then**7.         $symbols_{frame}=N/4$;8.       **end if**
9.   **Else**
10.      if OOFDM is ASCOor OOFDM is two LACO
**then**
11.         $symbols_{frame}=3N/4$;12.       **end if**
13. **end if**14. TX=[ ], $PAPR_{TOTAL}=\left[\;\right]$;15. ${\mathrm{bits}}_{\mathrm{size}}=K*symbols_{frame}*Z$; Total transmitted bits16. bits= **GenerateRandomBits** (${\mathrm{bits}}_{\mathrm{size}}$); Generated transmitted bits17. $Symbols=QAM_{\mathrm{mod}}\left(bits\right)$; Modulate the generated bits depend on the constellation order.18. $Symbols_{organised\;}=Matrix\left(Symbols,symbols_{frame},Z\right)$; Matrix form based on symbols number of the OOFDM approach and Z.19. for i=1:Z  do       $X_{S}=Symbols_{organised\;}\left(symbols_{frame},i\right)$; Select the transmitted symbols for each frame20.        **for** $\;m=1:{\mathrm{symbols}}_{\mathrm{frame}}$21.            **for** $n=1:{\mathrm{symbols}}_{\mathrm{frame}}$22.                $pc_{mn}\left(m,n\right)=\left(\mathrm{sqrt}\left(\frac{2}{{\mathrm{symbols}}_{\mathrm{frame}}+1}\right)\right)*\left(\cos\left(\left(\frac{\mathrm{pi}}{{\mathrm{symbols}}_{\mathrm{frame}}+1}\right)*\left(m-1\right)*\left(n-1\right)\right)\right)$;23.            **end for**
24.        **end for**
25.        $X_{precode}={\left[X_{S}{\;}^{T}*pc_{mn}\right]}^{T}$; Preceded modulated symbols26.        $X_{H}=Symbols_{arrangement}\left(X_{precode}\right)$; Symbols arrangement based on OOFDM approach as described in Section 327.        $x=IFFT\left(X_{H}\right)$, Convert to time domain28.        if OOFDM is DCO or OOFDM is ADO  then29.             $x_{clipped}={\left\lfloor x+F_{DC}\right\rfloor }_{c}\;$, $F_{DC}$ the dc level and $x_{clipped}$ time domain zero clipped signal30.           **else**
31.              if OOFDM is ACO or OOFDM is FLIP or OOFDM is ASCOor OOFDM is LACO 
**then**
32.                $x_{clipped}={\left\lfloor x\right\rfloor }_{c}$
33.              **end if**
34.        **end if**
35.        $x_{cp}=x_{clipped}+CP$, Add cyclic prefix36.        $x_{peak}=max\left({\left|x_{cp}\right|}^{2}\right)$, Peak power calculation37.        $x_{avg}=mean\left({\left|x_{cp}\right|}^{2}\right)$, Average power calculation38.        $PAPR=10*log_{10}\left(x_{peak}/x_{avg}\right)$, PAPR calculation39. $\;\;\;\;TX=\left[TX\;x_{cp}\right]$; → Transmitted signal40. $\;\;\;\;PAPR_{TOTAL}=\left[PAPR_{TOTAL}\;PAPR\right]$; → PAPR for the transmitted signal41. **end for**42. $\left[\mathrm{cdf},\;\mathrm{papr}\right]=ecdf\left(PAPR_{TOTAL}\right)$; → Calculate the complementary cumulative distribution function

**Algorithm 2.** Receiver emulation procedures.1. **for** *j* =1: length(snr) **do**2.     Output = [ ];3.     rx = TX + noise; → Received under AWGN channel4.     **for** *c* = 1: Z **do**5.           $r_{cp}$ = Remove **CP** (rx (: , c)); → Remove cyclic prefix6.           if OOFDM is DCO or OOFDM is ADO 
**then**
7.                 $rx_{clipped}=r_{cp}-F_{DC}$; → Remove dc shift8.               **else**
9.               if OOFDM is ACO or OOFDM is FLIP or OOFDM is ASCO  or OOFDM is LACO 
**then**
10.                   $rx_{clipped}=r_{cp}$;11.               **end if**
12.           **end if**
13.           if OOFDM is DCO or OOFDM is ADO 
**then**
14.                  $RX{\;}_{precoded}$ = **FFT**($rx_{clipped}$); → Convert to frequency domain15.                **else**
16.                if OOFDM is ACO or OOFDM is FLIP or OOFDM is ASCO  or OOFDM is LACO 
**then**
17.                        $RX_{precoded}$ = 2*FFT($rx_{clipped}$); → Convert to frequency domain18.                   **end if**
19.             **end if**
20.             $RX{\;}_{\mathrm{Symbols}}\;$= Detected symbols $\left(RX{\;}_{precoded}\right)$; → Detected symbols depend on data arrangement of the OOFDM approach use21.             $parallel_{symbols}={[{RX_{\mathrm{Symbols}}}^{T}*{pc_{mn}}^{-1}]}^{T}$; → Invers precoding matrix of PAPR reduction technique22.    $\;\;\;\;Serial_{symbols}=ParallelToSerial\left(parallel_{symbols}\right)\;$; → Convert to serial23.             Data = $QAM_{\mathrm{demod}}\left(Serial_{symbols}\right)$; → Demodulate the received symbols24.             Output = [ Output Data]; → Total Received bits25.    **end for**
26.    Error = BER (bits, Output); → BER calculation27. 
**end for**


## 5. Simulation Results and Discussion

In this part, we delve into the numerical simulations and outcomes concerning the multiple O-OFDM approaches designed for VLC communication systems, incorporating the novel precoding technique known as VLM. 

The received time-domain signal after using one of the previously mentioned approaches can described as follows [8]:

(37) $$y=gx_{OOFDM}+n,$$

where y is the received time-domain signal, $x_{OOFDM}$ is the transmitted time-domain signal, and g is the total channel gain, which can be described as g=ηRh; η is the electrical-to-optical efficiency of the LED. R is the responsivity of the photodetector (PD), and h is the optical channel gain, which is expressed as follows [53]:

(38) $$h=\frac{\left(m+1\right)A\;T\left(\psi \right)\;g\left(\psi \right)}{2\pi d^{2}}\;cos^{m}\left(\varphi \right)\cos\left(\psi \right),$$

where m is the Lambertian emission order, and A is the photodetector area. T(ψ) is the optical filter gain, and $g\left(\psi \right)=r^{2}/sin^{2}\left(\psi \right)$ is the gain of the optical concentrator with a refractive index r. ψ and ϕ denote the incident and emission angles, respectively. d is the distance between the transmitter and the receiver. The Lambertian order m can be calculated by

(39) $$m=\frac{-1}{log_{2}\left(cos\phi_{1/2}\right)}\;,$$

where $\phi_{1/2}$ is the half power angle of LED. The noise n follows the following Gaussian probability distribution function [15]:

(40) $$p\left(n\right)=\frac{1}{\sqrt{2\pi \sigma^{2}}}exp\left(\frac{-{\left(n-µ\right)}^{2}}{2\sigma^{2}}\right),$$

where µ and $\sigma^{2}$ are the mean and the variance, respectively. Here, we assume µ=0 and $\sigma^{2}=N_{0}/2$, and we assume that g is known at the receiver. Hence, the received symbol after equalization is:

(41) $$\tilde{y}=x_{OOFDM}+\tilde{n},$$

where $\tilde{n}=n/g$ is the additive Gaussian noise scaled by the total channel gain. To simplify the notation, we let $\tilde{y}$ denote the equalized received symbol in the simulation results. Therefore, the simulation results are obtained by assuming the communication channel is the AWGN channel as [2,3,9,16,18,20,22,24,41,42,48,49,51]. 

We have employed the O-OFDM approaches mentioned earlier using Matlab R2016b (9.1) software. Our simulations adhere to the parameters outlined in Table 5, with the AWGN channel serving as the communication medium between the sender and recipient. To evaluate the influence of modulation on system performance, we analyzed the simulation results for several constellation orders. For DCO and ADO-OFDM systems, DC-biasing levels of 7, 10, and 13 dB were utilized in the simulation to assess the impact of DC biasing on system performance. We began by examining the BER and PAPR performance of these O-OFDM schemes, comparing them under identical constellation orders. Subsequently, we extended our analysis to compare their performance at the same SE.

### 5.1. Exploring BER and PAPR for DCO-OFDM

**Figure 11:**
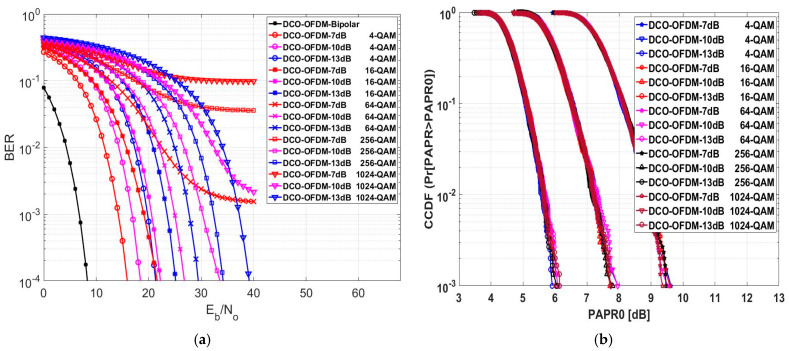
Performance of DCO-OFDM for (**a**) BER and (**b**) PAPR.

The BER of the DCO-OFDM system is evaluated and depicted in Figure 11a. When examining constellation sizes of 4-QAM and 16-QAM, it becomes clear that the 10 dB and 13 dB DC bias levels consistently demonstrated poorer BER performance than the 7 dB DC bias level. However, for higher constellation sizes, it is worth noting that the 10 dB and 13 dB DC bias levels delivered improved BER performance compared to the 7 dB DC bias level. Upon comprehensive analysis of the results, it is evident that the 10 dB DC bias level consistently outperformed the 13 dB level across the 16, 64, and 256 modulation orders. Consequently, it is advisable to utilize a 7 dB DC bias level when dealing with small constellation sizes, while a 13 dB DC bias level is more suitable for high constellation sizes like 1024-QAM. The DCO-OFDM system operating at 7 dB with a 16-QAM modulation scheme provided nearly the same BER performance when working at 10 dB with 4-QAM. Furthermore, the DCO-OFDM system working at 10 dB with a 256-QAM modulation scheme exhibited slightly better BER performance when operating at 13 dB with the same 256-QAM scheme. Finally, the numerical results for this approach are illustrated in Table 6 at a BER equal to $10^{-4}$ for various modulation schemes. 

Also, Figure 11a illustrates the effectiveness of changing the DC-biasing with different modulation orders in the DCO-OFDM system performance. For example, when employing high modulation orders, such as 512-QAM, and using low DC-biasing levels, such as 7 and 10 dB, the system’s performance was notably subpar, with a high bit error rate of $10^{-3}$ even for high SNR levels. However, the system exhibited superior performance when employing the same high modulation order but with a higher DC shift of 13 dB. However, lower modulation orders, such as 4-QAM, and low DC-biasing levels, such as 7 dB, performed better than 10 and 13 dB. This means that higher DC-biasing is needed for higher modulation orders to reduce the effect of zero clipping distortion because, in higher modulation orders, the Euclidean distance between any two adjacent symbols is lower than in lower modulation orders.

After evaluating the influence of DC biasing and modulation order on the system, we determined whether the PAPR was affected by both DC biasing and the selected modulation order. The CCDF of the DCO-OFDM system was assessed, as depicted in Figure 11b. At CCDF = $10^{-3}$, the value of PAPR0 for various modulation orders at a 13 dB DC bias level yielded superior evaluation results compared to the 10 dB and 7 dB DC bias levels. At a 13 dB DC bias level, the DCO-OFDM with a 4-QAM modulation yielded a lower PAPR0 value of 5.91 dB and increased to 6.14 dB with a 1024 modulation order. The DCO-OFDM with a 16-QAM modulation produced a lower PAPR0 value of 7.71 dB at a 10 dB DC bias level, but under the same bias condition, it increased to 7.962 with a 4-QAM modulation order. At a 7 dB DC bias level, the DCO-OFDM with a 1024-QAM modulation yielded a lower PAPR0 value of 9.36; however, a 4-QAM modulation order provided 9.625 dB. The average PAPR0 value across different modulation orders was 6.034 dB at a 13 dB DC bias, 7.834 dB at 10 dB, and 9.51 dB at 7 dB. According to this, the DC bias greatly affected the value of PAPR0 in DCO-OFDM. Still, for the DCO-OFDM, changing the modulation order did not result in a meaningful change in PAPR0.

### 5.2. Exploring BER and PAPR for ACO-OFDM

**Figure 12:**
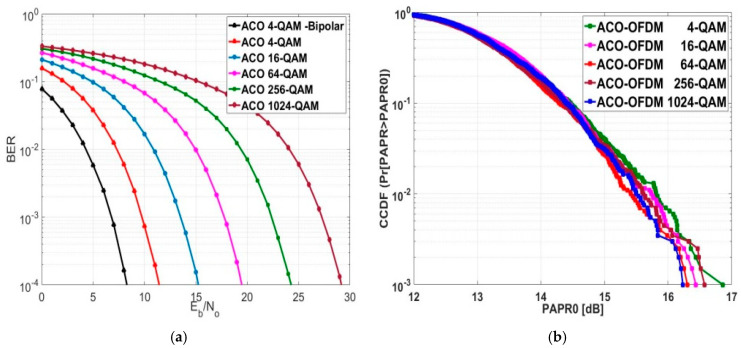
Performance of ACO-OFDM for (**a**) BER and (**b**) PAPR.

Figure 12a explores the BER assessment for ACO-OFDM. This scheme does not require a DC bias level like DCO-OFDM. However, for $\mathrm{BER}=10^{-4}$ with a 4-QAM modulation order, the $E_{b}/N_{O}$ for unipolar ACO decreased by 3.16 dB compared to bipolar ACO, mainly due to the clipping effect for transmitting the data via LEDs. For that reason, the primary focus of this research was on the thorough analysis and assessment of O-OFDM systems. This means analyzing the final broadcast and received signals and the system overall and accounting for the effects of clipping noise. As the modulation order of ACO-OFDM increased as the transmitted data rate increased, $E_{b}/N_{O}$ performance deteriorated. At a $\mathrm{BER}=10^{-4}$, the $E_{b}/N_{O}$ decreased by an average value of approximately 4.4325 dB when transitioning from one modulation order to another.

The CCDF of ACO-OFDM is depicted in Figure 12b. Table 6 illustrates the PAPR0 values of ACO-OFDM for various modulation orders at a CCDF of $10^{-3}$. The minimum PAPR0 value was attained at a CCDF of $10^{-3}$ with a 1024-QAM modulation order, while the maximum PAPR0 value was observed with a 4-QAM modulation order. The average PAPR0 value, computed from multiple evaluation data points, was 16.48 dB, while the PAPR0 median value was 16.44 dB at the 16-QAM modulation order.

### 5.3. Exploring BER and PAPR for FLIP-OFDM

**Figure 13:**
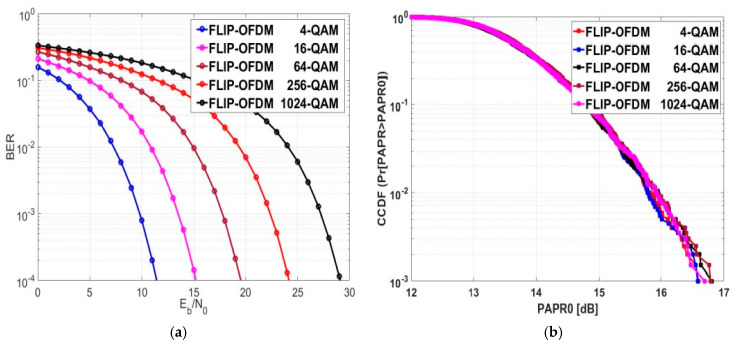
Performance of FLIP-OFDM for (**a**) BER and (**b**) PAPR.

The BER evaluation for FLIP-OFDM is displayed in Figure 13a. When the transmission data rate of FLIP-OFDM rose, the $E_{b}/N_{O}$ performance decreased. On average, there was a reduction of 4.4225 dB in $E_{b}/N_{O}$ when transitioning between modulation orders at a BER of $10^{-4}$. The numerical results presented in Table 6 indicate that, at different constellation sizes, the $E_{b}/N_{O}$ values for FLIP and ACO-OFDM were nearly the same. 

Figure 13b illustrates the CCDF performance of FLIP-OFDM. The resemblance in $E_{b}/N_{O}$ values between FLIP-OFDM and ACO-OFDM for various modulation orders suggests that their PAPR0 values are nearly identical. Additionally, it is worth noting that the PAPR0 of FLIP-OFDM did not exhibit significant changes as the modulation order was varied. The numerical values of PAPR0 at a CCDF of $10^{-3}$ for various modulation orders are provided in Table 6. For FLIP-OFDM evaluation results at a CCDF of $10^{-3}$, 16.69 dB was the median PAPR0 value for the 1024-QAM modulation order.

### 5.4. Exploring BER and PAPR for ADO-OFDM

**Figure 14:**
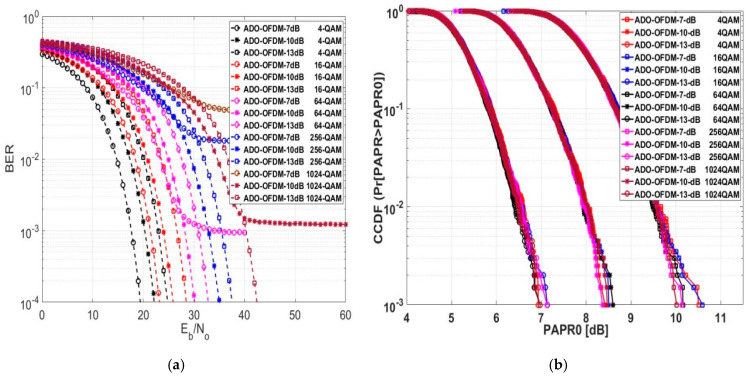
Performance of ADO-OFDM for (**a**) BER and (**b**) PAPR.

In Figure 14a, the ADO-OFDM system’s BER is assessed and displayed. ADO-OFDM, similar to DCO-OFDM, requires a DC bias value before clipping the time sampled signal before transmitting through LEDs. Hence, the performance of ADO-OFDM varies with alterations in the DC bias value. We assessed the BER performance at 7 dB, 10 dB, and 13 dB DC bias values. At a BER of $10^{-4}$, ADO-OFDM demonstrated lower $E_{b}/N_{o}$ for both the 4- and 16-QAM schemes at a 7 dB DC bias value compared to the performance at the same modulation schemes but with 10 and 13 dB DC bias values. However, $E_{b}/N_{o}$ at 10 dB and 13 dB DC bias values for other modulation schemes provided lower values comparable to $E_{b}/N_{o}$ at a 7 dB DC bias value. ADO-OFDM with a 13 dB DC bias value for 1024-QAM exhibited a lower $E_{b}/N_{o}$ compared to ADO-OFDM operating at a 10 dB DC bias value for the same 1024-QAM modulation. Otherwise, for other modulation schemes, ADO-OFDM with a 10 dB DC bias value showed an $E_{b}/N_{o}$ that is approximately 2.6 lower compared to ADO-OFDM operating at a 13 dB DC bias value. The numerical results for this scheme are presented in Table 6, showcasing various DC bias levels with different modulation orders.

At a CCDF of $10^{-3}$, the PAPR values of ADO-OFDM operating at a 13 dB DC bias level for various modulation orders showed lower values compared to ADO-OFDM operating at 10 and 7 dB DC bias values, as outlined in Figure 14b. Table 6 illustrates the comparison of PAPR0 values for different DC bias levels in ADO-OFDM across various modulation orders. The average PAPR0 values were 7.032 dB, 8.4706 dB, and 10.278 dB at the 13 dB, 10 dB, and 7 dB DC bias values, respectively. Based on the evaluated numerical data for DCO and ADO-OFDM, it was observed that the PAPR0 of ADO-OFDM was higher than that of DCO-OFDM at various DC bias levels across different modulation orders. It indicates that the dynamic range of the PAPR0 for DCO-OFDM was lower than that of ADO-OFDM for various DC bias levels and modulation orders by approximately an average value equal to 0.8 dB.

### 5.5. Exploring BER and PAPR for ASCO-OFDM

**Figure 15:**
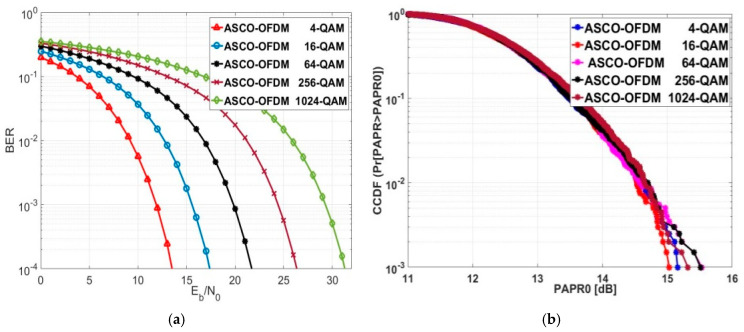
Performance of ASCO-OFDM for (**a**) BER and (**b**) PAPR.

The evaluated $E_{b}/N_{o}$ values for ASCO-OFDM decreased as the modulation order increased, showing a decreasing trend by an approximate average of 4.2, as depicted in Figure 15a. The corresponding numerical values for this evaluation are detailed in Table 6, specifically at a BER of $10^{-4}$. As previously mentioned, while the SE of ASCO-OFDM surpassed that of FLIP and ACO-OFDM, the $E_{b}/N_{o}$ evaluation values for both FLIP and ACO-OFDM were lower than those of ASCO-OFDM across different modulation orders, with an approximate difference of 2.1 dB at a BER of $10^{-4}$.

ASCO-OFDM’s CCDF is represented in Figure 15b. At a CCDF of $10^{-3}$, the PAPR0 values for ASCO-OFDM operating with 64-QAM and 256-QAM were approximately equal, measuring around 15.5 dB. The PAPR0 values for 4-QAM, 16-QAM, and 1024-QAM were recorded as 15.15 dB, 15.03 dB, and 15.32 dB, respectively. With various modulation orders, the average PAPR0 value of ASCO-OFDM was 15.306 dB. The ASCO-OFDM not only exhibited significantly higher SE values compared to FLIP and ACO-OFDM but also showcased lower PAPR values in contrast to both FLIP and ACO-OFDM. The approximate average reduction values were 1.174 dB for ACO-OFDM and 1.38 dB for FLIP-OFDM, respectively.

### 5.6. Exploring BER and PAPR for LACO-OFDM

**Figure 16:**
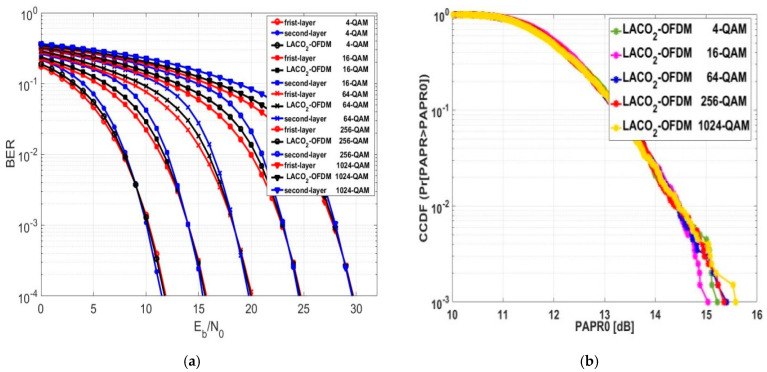
Performance of the two-layer LACO-OFDM for (**a**) BER and (**b**) PAPR.

As previously detailed in Section 3, the SE of LACO-OFDM is influenced upon the number of layers employed in its construction. Consequently, in this section, we aimed to evaluate the BER and PAPR for LACO-OFDM across various construction layers and different modulation orders. In Figure 16a, the BER performance of two-layer LACO-OFDM approach, denoted as ${\mathrm{LACO}}_{2}$-OFDM, is examined across different modulation orders. The numerical results indicate that the behavior of ${\mathrm{LACO}}_{2}$-OFDM was superior to that of ASCO-OFDM, despite both having the same SE. The $E_{b}/N_{o}$ values at a BER of $10^{-4}$ for ${\mathrm{LACO}}_{2}$-OFDM were, on average, approximately 1.7 dB lower than ASCO-OFDM across different modulation orders. However, the $E_{b}/N_{o}$ values for ${\mathrm{LACO}}_{2}$-OFDM were, on average, approximately 0.4 dB higher than FLIP and ACO-OFDM across different modulation orders. 

Figure 16b examines the CCDF performance of ${\mathrm{LACO}}_{2}$-OFDM across various modulation orders. With the modulation order changing, a minor change was observed in the PAPR0. At a CCDF of $10^{-3}$, the average PAPR0 value of ${\mathrm{LACO}}_{2}$-OFDM was the same as that of ASCO-OFDM, approximately equaling 15.3 dB. The PAPR0 values for LACO_2-OFDM using 64-QAM and 256-QAM were approximately similar to those provided by ASCO-OFDM.

**Figure 17:**
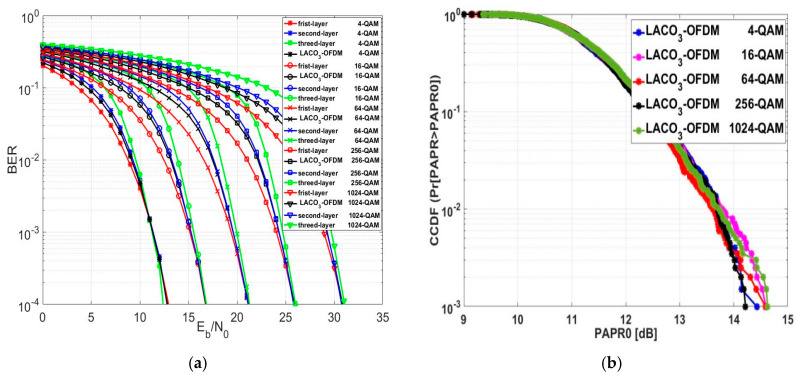
Performance of the three-layer LACO-OFDM for (**a**) BER and (**b**) PAPR.

The performance of the BER for the three-layer LACO-OFDM approach, referred to as ${\mathrm{LACO}}_{3}$-OFDM, is examined across different modulation orders in Figure 17a. The SE of ${\mathrm{LACO}}_{3}$-OFDM was higher than that of ASCO and ${\mathrm{LACO}}_{2}$-OFDM. However, the $E_{b}/N_{O}$ values of ${\mathrm{LACO}}_{3}$-OFDM at a BER of $10^{-4}$ were lower than those of ASCO-OFDM by an approximate average value of 0.544 dB but higher than those of FLIP, ${\mathrm{LACO}}_{2}$-OFDM, and ACO-OFDM by an approximate average value of 1.168 dB, 1.584 dB and 1.616 dB, respectively. 

Figure 17b explores the CCDF evaluation of ${\mathrm{LACO}}_{3}$-OFDM for different modulation orders. A slight change in the PAPR0 was noticed as the modulation order changed. At a CCDF of $10^{-3}$, the PAPR0 values of ${\mathrm{LACO}}_{3}$-OFDM with 16, 64, and 1024-QAM were approximately equal. The average PAPR0 value of ${\mathrm{LACO}}_{3}$-OFDM was approximately 0.813 dB lower than ASCO-OFDM, 1.987 dB lower than ACO-OFDM, 2.195 dB lower than FLIP-OFDM, and 0.827 dB lower than ${\mathrm{LACO}}_{2}$-OFDM. The evaluated PAPR0 results for ${\mathrm{LACO}}_{3}$-OFDM are presented in Table 6, showcasing the performance across various modulation orders.

**Figure 18:**
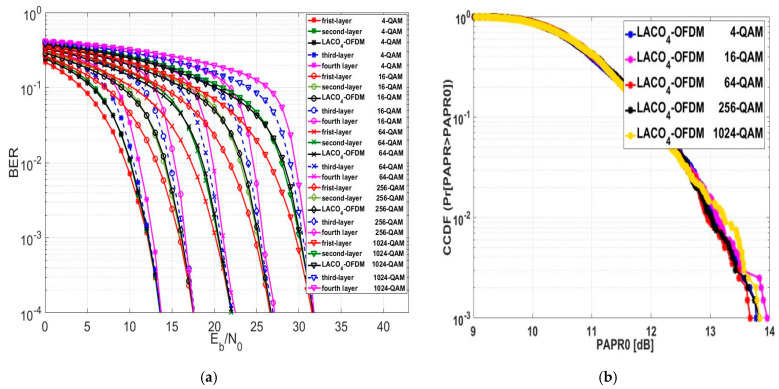
Performance of the four-layer LACO-OFDM for (**a**) BER and (**b**) PAPR.

Following the assessment of ${\mathrm{LACO}}_{2}$-OFDM and ${\mathrm{LACO}}_{3}$-OFDM, we moved forward to assess the LACO-OFDM approach with four layers, known as ${\mathrm{LACO}}_{4}$-OFDM. Figure 18a displays the BER performance of ${\mathrm{LACO}}_{4}$-OFDM. The evaluation was conducted across varying modulation orders, specifically within the ${\mathrm{LACO}}_{4}$-OFDM framework. The numerical results from the evaluation indicate that the BER performance of ${\mathrm{LACO}}_{4}$-OFDM was lower compared to ${\mathrm{LACO}}_{3}$, ${\mathrm{LACO}}_{2}$, ASCO, FLIP, and ACO-OFDM across various modulation orders. The average $E_{b}/N_{o}$ value for ${\mathrm{LACO}}_{4}$-OFDM at a BER of $10^{-4}$ exceeded that of ${\mathrm{LACO}}_{3}$, ${\mathrm{LACO}}_{2}$, ASCO, FLIP, and ACO-OFDM by approximately 0.824 dB, 10,992 dB, 0.28 dB, 2.408 dB, and 2.44 dB, respectively.

As we evaluated the BER performance of ${\mathrm{LACO}}_{4}$-OFDM, we also assessed the CCDF, which is illustrated in Figure 18b. At a CCDF of $10^{-3}$, the PAPR0 values for 4, 256, and 1024-QAM modulation orders were approximately equal. The PAPR performance of ${\mathrm{LACO}}_{4}$-OFDM was better compared to ${\mathrm{LACO}}_{3}$, ${\mathrm{LACO}}_{2}$, ASCO, FLIP, and ACO-OFDM across various modulation orders. At a CCDF of $10^{-3}$, the average PAPR0 value for ${\mathrm{LACO}}_{4}$-OFDM was less than that of ${\mathrm{LACO}}_{3}$, ${\mathrm{LACO}}_{2}$, ASCO, FLIP, and ACO-OFDM by around 0.6796 dB, 1.5066 dB, 1.4926 dB, 2.666 dB, and 2.8746 dB, correspondingly.

### 5.7. Investigating the Relationship between Spectral Efficiency and BER for Various O-OFDM Methodologies

**Figure 19:**
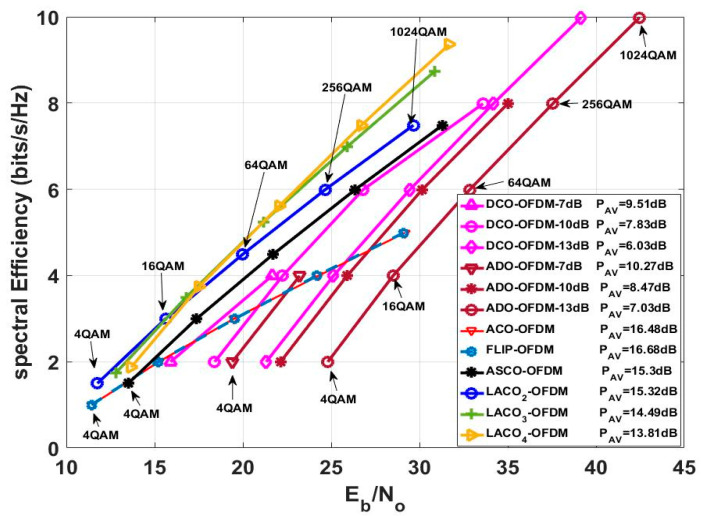
The $E_{b}/N_{o}$ needed for distinct levels of spectral efficiency at a BER of 10^−4^ for the DCO-OFDM, ACO-OFDM, FLIP-OFDM, ADO-OFDM, ASCO-OFDM, and LACO-OFDM techniques, considering alterations in constellation size.

After thoroughly evaluating the various factors affecting the choice of MCM techniques previously discussed, these elements are amalgamated in Figure 19. This visual depiction showcases the relationship between the spectral efficiency of different MCM techniques, their respective $E_{b}/N_{O}$ across diverse constellation sizes, and the average PAPR demonstrated by these techniques. The numerical outcomes derived from both Table 6 have been translated into a visual format and are presented in Figure 19. 

Both the ACO-OFDM and FLIP-OFDM approaches yielded similar $E_{b}/N_{O}$ values across various spectral efficiencies at different modulation orders and demonstrated equivalent average PAPR values. Furthermore, due to the rapid increase in $E_{b}/N_{O}$ as spectral efficiencies increased, it indicates that these two approaches are characterized as spectrally inefficient methods. The ASCO-OFDM and ${\mathrm{LACO}}_{2}$-OFDM approaches delivered identical spectral efficiency and average PAPR values, although ${\mathrm{LACO}}_{2}$-OFDM offered lower $E_{b}/N_{0}$ values across different modulation orders. The ADO-OFDM and DCO-OFDM methods yielded increased spectral efficiency values compared to other approaches, yet they also entailed higher $E_{b}/N_{O}$ values at equivalent modulation orders. Moreover, both methods presented varying average PAPR values associated with the chosen DC bias level. The DCO-OFDM approach exhibited superior BER and PAPR performance compared to the ADO-OFDM approach for various modulation orders at different DC bias levels. The spectral efficiency, $E_{b}/N_{O}$ values, and PAPR values offered by LACO-OFDM varied according to the number of layers employed in the technique. With an increase in the number of layers, there was a corresponding rise in spectral efficiency and $E_{b}/N_{O}$ values. However, this escalation was accompanied by a higher computational complexity. Interestingly, despite these increments, the average PAPR value decreased.

### 5.8. Investigating the Influence of VLM Precoding Methodology on BER and PAPR for Different O-OFDM Approaches

After conducting an extensive analysis of the BER and PAPR performance for MCM techniques used in VLC systems, a pressing need arose to explore a novel approach. This evaluation is essential to address the inherent constraints in these systems, with a particular emphasis on alleviating nonlinearity effects. The purpose of this section is to underscore the importance of the new technique by investigating its impact on BER and PAPR performance in comparison to the existing MCM approaches. 

Additionally, this work introduces two parameters, ${\mathrm{PAPR}}_{\mathrm{reduction}}$ and $E_{b}∕{N_{0}}_{\mathrm{difference}}$, for comparative analysis. ${\mathrm{PAPR}}_{\mathrm{reduction}}$ is characterized as the decrease in PAPR0 of the new approach relative to the conventional PAPR0. This is computed mathematically by subtracting the PAPR0 of the proposed methodology from the conventional PAPR0 for different MCM approaches at a CCDF of $10^{-3}$. A higher ${\mathrm{PAPR}}_{\mathrm{reduction}}$ value signifies that a more effective PAPR reduction technique has been utilized.

(42) $$PAPR_{reduction}=PAPR0_{conventional}-PAPR0_{proposed},$$

The parameter $E_{b}∕{N_{0}}_{\mathrm{difference}}$, on the other hand, is defined as the disparity between the conventional and the newly $E_{b}∕N_{o}$ after applying the PAPR degradation methodology. $E_{b}∕{N_{0}}_{\mathrm{difference}}$ is computed by subtracting the $E_{b}∕N_{o}$ value of the proposed methodology from the conventional value at a BER of $10^{-4}$. It is essential to note that there are three scenarios for the $E_{b}∕{N_{0}}_{\mathrm{difference}}$, value: When $E_{b}∕{N_{0}}_{\mathrm{difference}}$ is less than zero, the system’s BER performance is enhanced as it requires less energy to achieve the same BER. When $E_{b}∕{N_{0}}_{\mathrm{difference}}$ equals zero, there is no diminish in the BER performance. When $E_{b}∕{N_{0}}_{\mathrm{difference}}$ is greater than zero, the BER performance deteriorates because more energy is needed to attain the same BER.

(43) $$E_{b}∕{N_{0}}_{difference}=E_{b}∕{N_{0}}_{proposed}-E_{b}∕{N_{0}}_{conventional},$$

**Figure 20:**
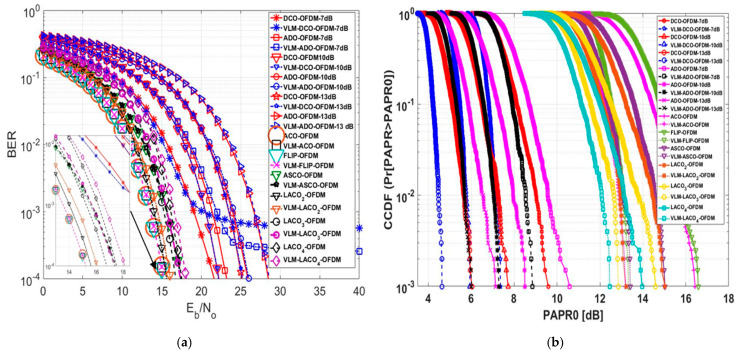
BER comparison between traditional O-OFDM approaches and proposed VLM-O-OFDM approaches using a 16-QAM modulation scheme for (**a**) BER and (**b**) PAPR.

In this assessment, a comprehensive analysis of the numerical outcomes was specifically conducted for the 16-QAM technique, as depicted in Figure 20 and Table 7. Figure 20a depicts the influence of employing VLM precoding methodology on the BER performance across various MCM approaches. At a BER of $10^{-4}$, the traditional ACO-OFDM $E_{b}/N_{0}$ value stood at 15.23 dB. Upon applying the VLM technique, there was no noticeable change in the $E_{b}/N_{0}$ value. Likewise, in the case of the FLIP-OFDM approach, employing the VLM precoding technique did not yield significant alterations in the $E_{b}/N_{0}$ value; the values remained relatively unchanged. The $E_{b}∕{N_{0}}_{\mathrm{difference}}$ for these two approaches was approximately zero, indicating no degradation in the BER performance for the FLIP-OFDM technique after the VLM precoding methodology was implemented.

The DC bias level significantly impacted the BER performance of DCO-OFDM and ADO-OFDM. At a 7 dB DC bias, these approaches could not be assessed at a BER of $10^{-4}$ after applying the VLM precoding technique but could be evaluated at a BER of $10^{-3}$. The $E_{b}∕{N_{0}}_{\mathrm{difference}}$ at a BER of $10^{-3}$ equalled 1 dB and 1.7 dB for ADO and DCO-OFDM, respectively. With a 10 dB DC bias, the $E_{b}∕{N_{0}}_{\mathrm{difference}}$ for DCO-OFDM was −0.6 dB, indicating a slight improvement in the BER performance of this approach. Conversely, $E_{b}∕{N_{0}}_{\mathrm{difference}}$ for ADO-OFDM at a 10 dB DC bias was 0.1 dB, signifying a slight degradation in the BER performance of this approach. At a 13 dB DC bias, the $E_{b}∕{N_{0}}_{\mathrm{difference}}$ for ADO and DCO-OFDM equalled 0.06 dB and 0.09 dB, almost approaching zero, suggesting no substantial degradation in the BER performance for these two approaches. For ASCO,${\;\mathrm{LACO}}_{2}$, ${\mathrm{LACO}}_{3}$, and ${\mathrm{LACO}}_{4}$-OFDM, the $E_{b}∕{N_{0}}_{\mathrm{difference}}$ values were 0.1 dB, 0.45 dB, 0.57 dB, and 0.6 dB, respectively. This indicates a slight degradation in the BER performance of these approaches. However, if the PAPR reduction is considerably greater than this degradation factor, this level of degradation might be acceptable. 

Figure 20b demonstrates that at a CCDF of $10^{-3}$, the conventional PAPR0 values for FLIP and ACO-OFDM were 16.44 dB and 15.58 dB, respectively. However, after adding the precoding technique, the proposed PAPR0 values were 13.17 dB and 13.32 dB, respectively. This resulted in PAPR0 reduction values of 3.27 dB for ACO-OFDM and 3.26 dB for FLIP-OFDM. Notably, the PAPR0 reduction values for FLIP and ACO -OFDM exceeded the reduction values for the other approaches, and the $E_{b}∕{N_{0}}_{\mathrm{difference}}$ for these two approaches was nearly zero.

At a 13 dB DC bias level, ADO and DCO-OFDM exhibited a PAPR reduction of 1.39 dB and 1.13 dB, respectively. Additionally, the $E_{b}∕{N_{0}}_{\mathrm{difference}}$ for these two approaches was nearly zero. When the DC bias level was set at 10 dB, DCO-OFDM showcased a PAPR reduction of 1.39 dB, while the $E_{b}∕{N_{0}}_{\mathrm{difference}}$ was −0.06 dB. At the same 10 dB DC bias level, ADO-OFDM revealed a PAPR reduction of 1.22 dB; however, this value changed to 1.73 dB at a 7 dB DC bias level, illustrating a variation in PAPR reduction concerning changes in the DC bias level. The PAPR reduction values were 1.62 dB, 1.81 dB, 1.735 dB, and 1.51 dB for ASCO, ${\mathrm{LACO}}_{2}$,${\;\mathrm{LACO}}_{3}$, and ${\mathrm{LACO}}_{4}$-OFDM, respectively. Table 6 presents the detailed numerical results of this assessment at a BER of $10^{-4}$ and a CCDF of $10^{-3}$. This evaluation highlights that the proposed PAPR methodology effectively reduces PAPR without causing any degradation in the BER performance for various MCM approaches.

**Figure 21:**
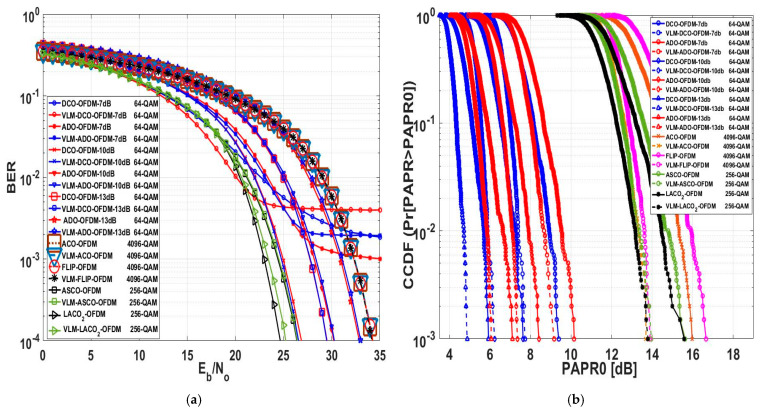
BER comparison between traditional O-OFDM approaches and proposed VLM-O-OFDM approaches at 5.9883 (bits/sec/Hz) spectral efficiency for (**a**) BER and (**b**) PAPR.

Figure 21a delves into assessing the BER performance of various O-OFDM approaches both pre- and post-implementation of the VLM methodology. These O-OFDM strategies maintained an identical spectral efficiency of 5.9883 (bits/sec/Hz). The LACO-OFDM performed better than other approaches by employing a smaller constellation size than FLIP and ACO-OFDM. It achieved this by avoiding utilising a DC bias level for clipping operations and transmitting the real unipolar signal within a single frame, unlike ASCO-OFDM, which relied on the concatenation of two frames. As mentioned earlier, DCO-OFDM demonstrated superior BER performance across various DC bias levels compared to ADO-OFDM. This performance advantage is attributed to the data arrangement within DCO-OFDM, which employs a single FFT/IFFT operation in both the transmitter and receiver. In contrast, ADO-OFDM employs dual FFT/IFFT operations in the transmitter, introducing noise from odd data subcarriers affecting the even subcarriers due to clipping effects. Additionally, the even data subcarriers in ADO-OFDM are impacted by clipping distortion caused by utilising the DC bias level in its process. DCO and ADO-OFDM delivered improved performance when operated at 10 dB and 13 dB DC bias levels compared to when operated at 7 dB. This enhancement is attributed to reduced clipping distortion as the DC bias level increases. The purpose of the clipping operation is to convert data into unipolar form. However, it is important to note that increasing the DC bias level beyond 13 dB may introduce upper clipping, resulting in signal distortion. At a BER of $10^{-4}$, the tabulated numerical results of this evaluation, comparing different modulation orders to attain an equivalent spectral efficiency for various O-OFDM approaches, both with and without the establishment of the VLM methodology, are presented in Table 8.

Figure 21b demonstrates the efficacy of the VLM methodology across various O-OFDM approaches and constellation sizes. At a CCDF of $10^{-3}$, ACO and FLIP-OFDM exhibited notably higher PAPR0 reduction values following the implementation of the VLM methodology compared to other O-OFDM approaches. Specifically, ACO-OFDM showed a PAPR0 reduction of 2.29 dB, while FLIP-OFDM achieved a reduction of 2.768 dB. In terms of $E_{b}∕{N_{0}}_{\mathrm{difference}}$, the ACO-OFDM approach demonstrated minimal change, almost zero, while the FLIP-OFDM approach slightly enhanced the $E_{b}/N_{0}$ performance by 0.107 dB at a BER = $10^{-4}$. The LACO-OFDM approach, after applying the VLM methodology, revealed a PAPR0 reduction of 1.79 dB at a CCDF of $10^{-3}$. However, there was a degradation in $E_{b}/N_{0}$ by 0.56 dB at a BER of $10^{-4}$. Table 8 presents the numerical results of the evaluation at a CCDF of $10^{-3}$, comparing different modulation orders to achieve an equivalent spectral efficiency across various O-OFDM approaches. It outlines the results both with and without the implementation of the VLM methodology.

## 6. Comparison with Related Studies

This section offers an in-depth comparative analysis between this study and the relevant literature, outlined in Table 9. The essential criteria for assessment and their respective values are elaborated upon in the previous sections. However, this study marks the initial exploration of the PAPR concept across various O-OFDM approaches. Table 9 highlights that this research presents a distinctive advantage by achieving the highest computed PAPR reduction value while maintaining the primary objective of minimizing the $E_{b}∕{N_{0}}_{difference}$ in all PAPR reduction-focused literature.

## 7. Conclusions

This paper introduces various Optical-OFDM techniques aimed at identifying an effective approach for VLC systems. The study introduces an innovative PAPR reduction methodology for various O-OFDM approaches, demonstrating minimal degradation in the BER. The PAPR reduction method was utilized to address the nonlinearity issue in O-OFDM. Notably, these reductions were achieved without any degradation in the system’s BER performance. The simulation results for different O-OFDM approaches demonstrate that as the modulation order increased, there was an enhancement in spectral efficiency and a decrease in BER performance. However, the PAPR performance remained relatively constant or approximately the same. Upon integrating the proposed method into various O-OFDM approaches, significant PAPR reduction values were achieved without noticeable degradation in BER. LACO-OFDM demonstrated superior BER performance compared to other O-OFDM approaches when operating under the same modulation scheme or spectral efficiency. ${\mathrm{LACO}}_{2}$-OFDM offered equivalent spectral efficiency and PAPR as ASCO-OFDM, yet with reduced computational complexity. Specifically, the PAPR values for ${\mathrm{LACO}}_{2}$-OFDM,${\;\mathrm{LACO}}_{3}$-OFDM, and ${\mathrm{LACO}}_{4}$-OFDM were 15.03 dB, 14.595 dB, and 13.96 dB, respectively. After the applied of the precoding methodology, the PAPR reduction values for these systems were observed as 1.81 dB, 1.735 dB, and 1.51 dB, respectively. Furthermore, the $E_{b}∕N_{o}$ values for ${\mathrm{LACO}}_{2}$-OFDM,${\;\mathrm{LACO}}_{3}$-OFDM, and ${\mathrm{LACO}}_{4}$-OFDM were identified as 15.62 dB, 16.78 dB, and 17.52 dB. However, the $E_{b}∕N_{o}$ for these systems degraded by values of 0.45 dB, 0.57 dB, and 0.6 dB, respectively. ACO-OFDM demonstrated a PAPR reduction of 3.27 dB, while FLIP-OFDM exhibited a reduction of 3.26 dB without any degradation in BER performance. DCO-OFDM demonstrated superior BER and PAPR performance compared to ADO-OFDM when operating under the same modulation scheme and DC bias level. This advantage is attributed to the information hierarchy inherent in DCO-OFDM. The research involves comparing related literature to validate and establish the novelty of the proposed schemes and assessing their performance within the field. Ultimately, this study advances the compatibility of various O-OFDM systems for practical applications.

## Figures and Tables

**Table 2:** The assessment of spectral efficiency (bits/sec/Hz) values across multiple O-OFDM approaches for varying FFT sizes using a 16-QAM modulation scheme.

MCM TECHNIQUE	N = 8	N = 16	N = 32	N = 64	N = 128	N = 256	N = 512	N = 1024	N = 2048	N = 4096
DCO	3.2	3.5556	3.7647	3.8788	3.9385	3.9690	3.9844	3.9922	3.9961	3.9980
ACO	1.6	1.7778	1.8824	1.9394	1.9692	1.9845	1.9922	1.9961	1.9980	1.9990
FLIP	1.6	1.7778	1.8824	1.9394	1.9692	1.9845	1.9922	1.9961	1.9980	1.9990
ASCO	2.4	2.6667	2.8235	2.9091	2.9538	2.9767	2.9883	2.9942	2.9971	2.9985
ADO	3.2	3.5556	3.7647	3.8788	3.9385	3.9690	3.9844	3.9922	3.9961	3.9980
LACO_2	2.4	2.6667	2.8235	2.9091	2.9538	2.9767	2.9883	2.9942	2.9971	2.9985
LACO_3	2.8	3.1111	3.2941	3.3939	3.4462	3.4729	3.4864	3.4932	3.4966	3.4983
LACO_4	3	3.3333	3.5294	3.6364	3.6923	3.7209	3.7354	3.7427	3.7463	3.7482

**Table 3:** The computational complexity of different O-OFDM approaches for both the transmitter and receiver components.

MCM TECHNIQUE	Complexity	TX	RX
DCO-OFDM	$2\;O({\mathrm{Nlog}}_{2}N)$	$O({\mathrm{Nlog}}_{2}N)$	$\;O({\mathrm{Nlog}}_{2}N)$
ACO-OFDM	$2\;O({\mathrm{Nlog}}_{2}N)$	$O({\mathrm{Nlog}}_{2}N)$	$\;O({\mathrm{Nlog}}_{2}N)$
FLIP-OFDM	$2\;O({\mathrm{Nlog}}_{2}N)$	$O({\mathrm{Nlog}}_{2}N)$	$\;O({\mathrm{Nlog}}_{2}N)$
ASCO-OFDM	$8\;O({\mathrm{Nlog}}_{2}N)$	$3\;O({\mathrm{Nlog}}_{2}N)$	$5\;O({\mathrm{Nlog}}_{2}N)$
ADO-OFDM	$5\;O({\mathrm{Nlog}}_{2}N)$	$2\;O({\mathrm{Nlog}}_{2}N)$	$3\;O({\mathrm{Nlog}}_{2}N)$
LACO,2-OFDM	$5\;O({\mathrm{Nlog}}_{2}N)$	$2\;O({\mathrm{Nlog}}_{2}N)$	$3\;O({\mathrm{Nlog}}_{2}N)$
LACO,3-OFDM	$8\;O({\mathrm{Nlog}}_{2}N)$	$3\;O({\mathrm{Nlog}}_{2}N)$	$5\;O({\mathrm{Nlog}}_{2}N)$
LACO,4-OFDM	$11\;O({\mathrm{Nlog}}_{2}N)$	$4\;O({\mathrm{Nlog}}_{2}N)$	$7\;O({\mathrm{Nlog}}_{2}N)$

**Table 4:** The specific numerical values for the addition and multiplication operations concerning the previously mentioned approaches while maintaining a constant FFT/IFFT size of 1024.

MCM TECHNIQUE	TX Complexity	TX ADD	TX MULT	RX Complexity	RX ADD	RX MULT
DCO-OFDM	$O({\mathrm{Nlog}}_{2}N)$	27,652	7172	$\;O({\mathrm{Nlog}}_{2}N)$	27,652	7172
ACO-OFDM	$O({\mathrm{Nlog}}_{2}N)$	27,652	7172	$\;O({\mathrm{Nlog}}_{2}N)$	27,652	7172
FLIP-OFDM	$O({\mathrm{Nlog}}_{2}N)$	27,652	7172	$\;O({\mathrm{Nlog}}_{2}N)$	27,652	7172
ASCO-OFDM	$3\;O({\mathrm{Nlog}}_{2}N)$	82,956	21,516	$5\;O({\mathrm{Nlog}}_{2}N)$	138,260	35,860
ADO-OFDM	$2\;O({\mathrm{Nlog}}_{2}N)$	55,304	14,344	$3\;O({\mathrm{Nlog}}_{2}N)$	82,956	21,516
LACO,2-OFDM	$2\;O({\mathrm{Nlog}}_{2}N)$	55,304	14,344	$3\;O({\mathrm{Nlog}}_{2}N)$	82,956	21,516
LACO,3-OFDM	$3\;O({\mathrm{Nlog}}_{2}N)$	82,956	21,516	$5\;O({\mathrm{Nlog}}_{2}N)$	138,260	35,860
LACO,4-OFDM	$4\;O({\mathrm{Nlog}}_{2}N)$	110,608	28,688	$7\;O({\mathrm{Nlog}}_{2}N)$	193,564	50,204

**Table 5:** Simulation parameters.

Parameter	Value
OOFDM symbols	1000
FFT/IFFT size	1024
Modulation technique	QAM
Constellation order	4, 16, 64, 256, 1024, 4096
DC-bias level	7,10,13
Cyclic prefix	1024/4 = 256
Channel model	AWGN

**Table 6:** The $E_{b}/N_{O}$ (dB) at a BER of $10^{-4}$ and PAPR0 (dB) at a CCDF of $10^{-3}$ numerical outcomes for different O-OFDM approaches across different constellation sizes.

MCM	4-QAM		16-QAM		64-QAM		256-QAM		1024-QAM	
	BER	PAPR0	BER	PAPR0	BER	PAPR0	BER	PAPR0	BER	PAPR0
DCO_7dB	15.9	9.625	21.65	9.58	NA	9.53	NA	9.455	NA	9.36
DCO_10dB	18.4	7.962	22.25	7.71	26.8	7.948	33.6	7.8	NA	7.75
DCO_13dB	21.32	5.91	25.13	6.02	29.45	6.07	34.18	6.03	39.15	6.14
ACO	11.44	16.86	15.23	16.44	19.48	16.3	24.28	16.57	29.17	16.23
FLIP	11.42	16.59	15.2	16.58	19.53	16.81	24.18	16.77	29.11	16.69
ASCO	13.51	15.15	17.37	15.03	21.71	15.52	26.35	15.51	31.3	15.32
ADO_7dB	19.4	10.5	23.22	10.59	NA	10.15	NA	10.13	NA	10.02
ADO_10dB	22.13	8.44	25.9	8.5	30.15	8.59	35	8.353	NA	8.47
ADO_13dB	24.81	6.94	28.5	7.14	32.84	6.97	37.55	7.13	42.46	6.98
**LACO_2_**	11.76	15.22	15.62	15.03	19.99	15.4	24.65	15.38	29.66	15.57
**LACO_3_**	12.82	14.43	16.78	14.595	21.16	14.59	25.9	14.22	30.86	14.63
**LACO_4_**	13.67	13.77	17.52	13.96	22.06	13.67	26.71	13.83	31.68	13.837

**Table 7:** PAPR0 and $E_{b}/N_{0}$ values at a CCDF of $10^{-3}$ and a BER of $10^{-4}$. Additionally, the PAPR reduction value and the disparity in $E_{b}/N_{0}$ between conventional O-OFDM approaches and the newly introduced O-OFDM approaches after integrating the VLM technique with a 16-QAM modulation scheme.

MCM Approach	$E_{b}/N_{O}$ Conventional (dB)	$E_{b}/N_{O}$ VLM (dB)	$E_{b}/N_{O}$ Difference (dB)	$PAPR_{0}$ Conventional (dB)	$PAPR_{0}$ VLM (dB)	PAPR Reduction (dB)
DCO-7 dB	21.65	N/A	N/A	9.58	7.36	2.22
ADO-7 dB	23.22	N/A	N/A	10.59	8.86	1.73
DCO-10 dB	22.25	22.19	−0.06 ≈ 0	7.71	5.94	1.77
ADO-10 dB	25.9	26	0.1 ≈ 0	8.5	7.28	1.22
DCO-13 dB	25.13	25.19	0.06 ≈ 0	6.02	4.63	1.39
ADO-13 dB	28.5	28.59	0.09 ≈ 0	7.14	6.01	1.13
ACO	15.23	15.24	0.01 ≈ 0	16.44	13.17	3.27
FLIP	15.2	15.23	0.03 ≈ 0	16.58	13.32	3.26
ASCO	17.37	17.47	0.1 ≈ 0	15.03	13.41	1.62
$LACO_{2}$	15.62	16.07	0.45	15.03	13.22	1.81
$LACO_{3}$	16.78	17.35	0.57	14.595	12.86	1.735
$LACO_{4}$	17.52	18.12	0.6	13.96	12.45	1.51

**Table 8:** PAPR0 and $E_{b}/N_{0}$ values at a CCDF of $10^{-3}$ and a BER of $10^{-4}$. Additionally, the PAPR reduction value and the disparity in $E_{b}/N_{0}$ between conventional O-OFDM approaches and the newly introduced O-OFDM approaches after integrating the VLM technique with different modulation scheme but at the same spectral efficiency of 5.9883 (bits/sec/Hz).

MCM Approach	Modulation Scheme	$E_{b}/N_{O}$ Conventional (dB)	$E_{b}/N_{O}$ VLM (dB)	$E_{b}/N_{O}$ Difference (dB)	$PAPR_{0}$ Conventional (dB)	$PAPR_{0}$ VLM (dB)	PAPR Reduction (dB)
DCO-7db	64-QAM	N/A	N/A	N.A	9.385	7.725	1.66
ADO-7db	64-QAM	N/A	N/A	N.A	10.154	9.159	0.995
DCO-10db	64-QAM	26.86	26.55	−0.31	7.633	6.23	1.403
ADO-10db	64-QAM	30.2	30.26	0.06 ≈ 0	8.411	7.357	1.054
DCO-13db	64-QAM	29.45	29.41	−0.04	5.93	4.87	1.06
ADO-13db	64-QAM	32.84	33	0.16 ≈ 0	7.11	6.06	1.05
ACO	4096-QAM	34.25	34.278	0.028 ≈ 0	15.97	13.68	2.29
FLIP	4096-QAM	34.277	34.17	−0.107	16.658	13.89	2.768
ASCO	256-QAM	26.35	26.42	0.07 ≈ 0	15.63	13.96	1.67
$LACO_{2}$	256-QAM	24.66	25.22	0.56	15.58	13.79	1.79

**Table 9:** Comparison between the novel proposed O-OFDM approaches and other approaches.

References	MCM Approach	FFT/IFFT Size	Modulation Scheme	PAPR Reduction (dB)	$E_{b}/N_{O}$ Difference (dB)
[3]	Pilot-Assisted Optical OFDM	1024	M-QAM	≈2.2 dB	N/A
[24]	*μ*-SOOFDM	64	16-QAM	0.45 1.93	≈0
[42]	ESACO OFDM	128	16-QAM 64-QAM 256-QAM	≈1.2	1 2 3
[49]	OFDM-based VLC with DCT	128	4-QAM	1.4	0
[51]	WHT-DCO-OFDM DHT-DCO-OFDM VLM-DCO-OFDM WHT-ACO-OFDM DHT-ACO-OFDM	256	16-QAM	0.54 0.99 1.87 0.93 1.27	N/A
[52]	WHT-precoding OOFDM DCT-precoding OOFDM	256	16-QAM	0.5	≈0
Proposed Techniques	VLM-ACO-OFDM VLM-FLIP-OFDM VLM-DCO-OFDM-10 dB VLM-ADO-OFDM-10 dB VLM-DCO-OFDM-13 dB VLM-ADO-OFDM-13 dB VLM-ASCO-OFDM VLM-LACO_2_-OFDM VLM-LACO_3_-OFDM VLM-LACO_4_-OFDM VLM-DCO-OFDM-10 dB VLM-ADO-OFDM-10 dB VLM-DCO-OFDM-13 dB VLM-ADO-OFDM-13 dB VLM-ASCO-OFDM VLM-LACO2-OFDM VLM-ACO-OFDM VLM-FLIP-OFDM	1024	16-QAM 16-QAM 16-QAM 16-QAM 16-QAM 16-QAM 16-QAM 16-QAM 16-QAM 16-QAM 64-QAM 64-QAM 64-QAM 64-QAM 256-QAM 256-QAM 4096-QAM 4096-QAM	3.27 3.26 1.77 1.22 1.39 1.13 1.62 1.81 1.735 1.51 1.403 1.054 1.06 1.05 1.67 1.79 2.29 2.768	≈0 ≈0 −0.06 ≈0 ≈0 ≈0 ≈0 0.45 0.57 0.6 −0.31 ≈0 −0.04 ≈0 ≈0 0.56 ≈0 −0.107

## Data Availability

All the data have been included in the study.
